# Zinc finger protein 703 induces EMT and sorafenib resistance in hepatocellular carcinoma by transactivating CLDN4 expression

**DOI:** 10.1038/s41419-020-2422-3

**Published:** 2020-04-08

**Authors:** Hao Wang, Hongfa Xu, Feng Ma, Meixiao Zhan, Xiangyu Yang, Shengni Hua, Wei Li, Yong Li, Ligong Lu

**Affiliations:** 1 Zhuhai Interventional Medical Center, Zhuhai Precision Medical Center, Zhuhai People’s Hospital, Zhuhai Hospital Affiliated with Jinan University, Jinan University, 519000 Zhuhai, Guangdong China; 2 School of Life Sciences, Tsinghua University, 100084 Beijing, China; 3 Department of Gastroenterology, The First Affiliated Hospital of Jinan University, 510630 Guangzhou, China

**Keywords:** Tumour biomarkers, Prognostic markers

## Abstract

Metastasis is one of the most common reasons of hepatocellular carcinoma (HCC) death; however, the molecular mechanism underlying HCC metastasis remains incompletely defined. Here we report a new function of Zinc Finger Protein 703 (ZNF703), a member of the NET/NlZ family of zinc finger transcription factors, in promoting hepatocellular carcinoma metastasis. We demonstrated that the overexpression of ZNF703 in human HCC tissue is correlated with tumor metastasis and recurrence, it is also related with the prognosis and survival rate of patients. ZNF703 overexpression promotes HCC progression in vitro and in vivo, whereas ZNF703 knockdown has the opposite effect. In addition, ZNF703 induces epithelialmesenchymal transition (EMT) via directly binding to the CLDN4 promoter and transactivating CLDN4 expression. Downregulation of CLDN4 can attenuate ZNF703-mediated HCC metastasis, whereas upregulation of CLDN4 can reverse the decreased metastasis induced by ZNF703 knockdown. Our data revealed that ZNF703 expression is correlated with CLDN4 level, the overexpression of both ZNF703 and CLDN4 are leaded to poorer prognosis of patients with HCC. Moreover, ZNF703 knockdown can enhance the sensitivity of HCC cell to sorafenib, whereas ZNF703 overexpression has the opposite effect. These results suggested that ZNF703 might be a potential target for cancer therapies and a candidate prognostic biomarker for predicting whether patients with HCC are befitting for sorafenib treatment.

## Introduction

Hepatocellular carcinoma (HCC) is the third leading cause of cancer-related mortality with nearly 700,000 annual deaths globally^1^. Although advances in surgical resection, which remains a potentially curative treatment, the prognosis of patients with HCC is still not optimistic^2^. Unfortunately, the majority of HCC patients are diagnosed at an advanced stage, and a radical treatment is not the most suitable option. The distant metastasis after surgical resection is the major reason for the poor prognosis of patients with HCC^3^. Nonetheless, the underlying molecular mechanism associated with HCC metastasis still remains unclear. Therefore, it is urgent to explore the mechanism of HCC metastasis.

ZNF703, which is a member of the NET/NlZ family of zinc finger transcription factors, is crucial for the embryonic development of zebrafish^4^, Xenopus^5^ and Drosophila^6^. Several recent studies reported that the aberrant expression of ZNF703 is involved in tumor progression. It has been shown that ZNF703 induces EMT through repressing E-cadherin expression and stimulates tumor migration and invasion in breast cancer^7^. In addition, high expression level of ZNF703 is associated with poorer prognosis in breast cancer patients^8–10^. A recent study also found that ZNF703 increases the migration and invasion of oral cancer cells by activating the PI3K/AKT/GSK-3β signaling-pathway and that ZNF703 overexpression is negatively correlated with overall survival in patients with oral squamous cell carcinoma (OSCC)^11^. These studies suggested that ZNF703 might promote tumor metastasis by promoting EMT.

CLDN4, a critical member of the claudin family, is widely expressed invarious types of tumors and contributes to cell migration and invasion^12–16^. It is well known that the claudin family play important roles in the constitution and maintenance of tight junctions^17^. Accumulating evidencehas indicated that abnormal expression of CLDN4 may lead to a tendency for cancer to metastasizemainly because CLDN4 could significantly increase cancer cells invasive properties and promote epithelial-mesenchymal transition (EMT). Previous studies reported that CLDN4 promotes EMT and cancer metastasis through activating MMP and ZEB family members^18–20^. By using RNA sequencing in the present study, we found that ZNF703 upregulates CLDN4 expression in HCC cells. Therefore, we hypothesized that ZNF703 promotes HCC metastasis by upregulatingCLDN4 expression.

To date, no studies have reported on the biological function and clinicopathologic significance of ZNF703 in HCC. In the present study, we present the first evidencethat ZNF703 overexpression is involved in HCCmetastasis and indicates pooroutcomes. ZNF703 facilitates HCC invasion and metastasis by inducing EMT by way of transactivating CLDN4 expression, which is subsequently shown to mediate this EMT and sorafenib resistance. Moreover, higher expressions of ZNF703 are also correlated with impaired sorafenib response and the poorer prognosis of HCC patients.

## Materials and methods

### Cell culture

The human HCC cell lines HCCLM3, MHCC97L, MHCC97H, SMMC7721, HepG2, Hep3B, Huh-7 and the normal hepatocyte cell line (LO2) were purchased from Cell resource center, Shanghai institute of life sciences, Chinese academy of sciences (Shanghai, China). TheHCCLM3, MHCC97L, MHCC97H and Huh-7 were cultured in DMEM (Invitrogen) with 10% fetal bovine serum (FCS; Hyclone, Invitrogen) and the SMMC-7721, HepG2, Hep3B, and LO2 were cultured in RPMI-1640 (Invitrogen) supplemented with 10% fetal bovine serum (Hyclone, Invitrogen). All cells were incubated in a humidified chamber with 5% CO2 at 37 °C.

### Patients and tissue specimens

All of the HCC and adjacent tissueswere collected at the time of diagnosis before any therapy from the People’s Hospital of Zhuhai City, China. This project was approved by the Ethical Review Committee of the People’s Hospital of Zhuhai City. Written informed consent was obtained from all the patients. The prognostic value of ZNF703 in HCC was further confirmed in The Cancer Genome Atlas (TCGA) dataset (http://www.cbioportal.org).

### Western blot

Western blot was performed, as we previously described^21^. The primary antibodies are as followings:Rabbit polyclonal to E-Cadherin (ab15148, Abcam), Rabbit polyclonal to N-Cadherin (ab18203, Abcam), Mousemonoclonalto Vimentin (ab8978, Abcam), Rabbit monoclonal to ZEB1 (ab203829, Abcam), Rabbit polyclonal to beta Actin (ab8227, Abcam), Rabbit polyclonal to ZNF703 (ab155210, Abcam) and Rabbit polyclonal to Claudin 4 (ab15104, Abcam). β-actin was served as an internal control.

### Real-time PCR

Real-time PCR was performed as we previously described^21^. The assessment of gene expression was done by using TB Green® Premix Ex Taq™ II (TliRNaseH Plus) (Takara) and Applied Biosystems 7300 Real-Time PCR System (Thermo Fisher Scientific), and primers are as followings: ZNF703 forward: 5′-GCTATTTGACGTGCGGCTC-3′, reverse: 5′-CACCGAGTTGAGTTTGGAGGA-3′; CLDN4 forward: 5′-CAAAGGTGCACTCTGCGAAC-3′, reverse: 5′-GATGGTCTGCGAGGTGACAA-3′; β-actin forward: 5′-TGGCACCCAGCACAATGAA-3′, reverse: 5′-CTAAGTCATAGTCCGCCTAGAAGCA-3′; β-actin was used as an endogenous control. Relative mRNA expression of ZNF703 or CLDN4 was calculated using the 2^−ΔΔCt^ method.

### Immunofluorescence assay

Immunofluorescent staining was performed as we previously described^21^. The primary antibodies are as followings:Rabbit polyclonal to ZNF703 (ab155210, Abcam), E-Cadherin (ab15148, Abcam) and Mouse monoclonal to Vimentin (ab8978, Abcam). The images were captured by an inverted fluorescence microscope (DMi8, leica).

### Immunohistochemistry

The immunohistochemistry analysis was performed, as we previously described^21^. The primary antibodies were used: Rabbit polyclonal to Ki67 (ab15580, 1:200, Abcam), Rabbit polyclonal to ZNF703 (ab155210, 1:100, Abcam) and Rabbit polyclonal to Claudin 4 (ab15104, 1:100, Abcam).

### Lentivirus constructs and stable cell lines

Lentivirus was purchased from GeneChem (Shanghai, China). The HCCLM3 were usedfor the downregulation assays, and SMMC-7721 cells were used for the upregulation assays according to the manufacturer’s protocol. Sequences for the shRNAs were as follows: shZNF703-1: 5′-GGTAGGTGACTCTCGCTAGAT-3′, shZNF703-2: 5′-GCTGTATGGACAGAGACTAGC-3′, shZNF703-3: 5′-GCGCTCACCTGGTTGAATTCA-3′, shCLDN4: 5′-GCTCAGGAATCCAGAGAAACT-3′. Stably transfected cells were selected by G418 for four weeks.

### Cell wound-healing, migration and invasion assays

Cell wound-healing, migration and invasion assayswere performed, as we previously described^21^. For the invasion assay, all the procedures were the same to that of the cell migration assay, except that transwell membranes were pre-coated with Matrigel (R&D Systems, USA).

### Cell proliferation

HCC cell proliferation was determined by CCK-8 assay (Beyotime, China). Cells were seeded in 96-well plates at a density of 5.0 × 10^3^ per well and treated with increasing doses of sorafenib. After culture for 48 h, 10 μl CCK-8 reagent was added to eachwell and incubated for 1 h. The absorbance value (OD) of each well was measured at 450 nm.

### Colony formation

HCC cells were seeded in 6-well plates at a density of 5.0 × 10^2^ per well and treated with sorafenib (2 μM). After incubation for 2 weeks, Colonies were fixed with methanol and stained with 0.5% crystal violet.

### RNA-sequencing analysis

Total RNA was extracted from the ZNF703 knockdown (shZNF703) and its negative control (shcontrol) groups of HCCLM3 cells using TRizol reagent (Invitrogen, California, USA). RNA-sequencing analysiswas carried out by Sinotech Genomics Co. Ltd. (Shanghai, China).

### Plasmid construction

Plasmid construction was performed according to the standard procedures in our previous study^21^. Primers were designed to clone construct of the (−1684/+120) CLDN4 promoter from human genomic DNA. This sequence was cloned intothe pGL3-Basic vector (Promega). The 5′-flanking deletion constructs of the CLDN4 promoter (−1242/+120) CLDN4 and (−751/+120) CLDN4 were similarly generated using the construct of (−1684/+120) CLDN4 promoter as the template. The QuikChangeII Site-Directed Mutagenesis Kit (Stratagene) was used to mutate the ZNF703-binding sites in the CLDN4 promoter. The constructs were validated by DNA sequencing. All of the primers are shown in supplementary Table S1.

### Luciferase reporter assay

The SMMC-7721 cells were co-transfected withpCMV-ZNF703 or the negative control, pGL3-CLDN4 promoter and Renilla luciferase expression vector (pRL-RSV).48 h after transfection, luciferase activity was detected by using the Dual Luciferase ReporterAssay System (Promega, CA, USA) according to the manufacturer’s instructions. The activity of firefly luciferase was normalized to that of Renilla luciferase level.

### Chromatin immunoprecipitation (ChIP) assay

Cells were collected 48 h after transfection and were cross-linked in 1% formaldehyde at 37 °C for 10 min. After washing, cells were resuspended in lysis buffer. Supernatants were sonicated, incubated with anti-ZNF703 (ab241069, Abcam) or control IgG at 4 °C overnight, immunoprecipitated at 4 °C for 2 h, and then captured by protein A/G beads (Sigma-Aldrich, USA). The precipitated DNA was purified, and quantified by using real-time PCR assay. All of the primers for ChIP are shown in supplementary Table S1.

### In vivo metastasis assays

HCC cells (2 × 10^6^ per mouse) were injected into the tail vein of BALB/C nude mice (4 weeks old). Eight weeks later, the mice were sacrificed, their lungs were dissected. The lungs then were fixed in 10% formalin and stained with hematoxylin and eosin (H&E). A dissecting microscope was conducted to count the numbers of lung metastases. Mice were housed under specific pathogen free (SPF) conditions. All animal experiments were approved by the Animal Experimentation Ethics Committee of the People’s Hospital of Zhuhai City, Guangdong, China.

### In vivo sorafenibsensitivity assay

HCC cells (2 × 10^6^) were subcutaneously injected into the flanks of BALB/C nude mice (4 weeks old). Two days after inoculation, mice were administered with oral sorafenib at the dose of 2.0 mg/kg every other day. Four weeks later, the mice were sacrificed, their tumors were removed and weight and Volumes of tumors were measured. The calculated formula of Volumes was: volume (v) = (length × width^2^)/2. All animal experiments were approved by the Animal Experimentation Ethics Committee of the People’s Hospital of Zhuhai City, Guangdong, China.

### Statistical analysis

Statistical analysis was performed by using SPSS 16.0 software (Chicago, IL, USA). The Kaplan-Meier method was used in the recurrence and survival data. All tests were two-tailed and differences were considered statistically significant when *p* < 0.05.

## Results

### ZNF703 expression is increased in HCC tissues and related with poor prognosis of patients

**Fig. 1:**
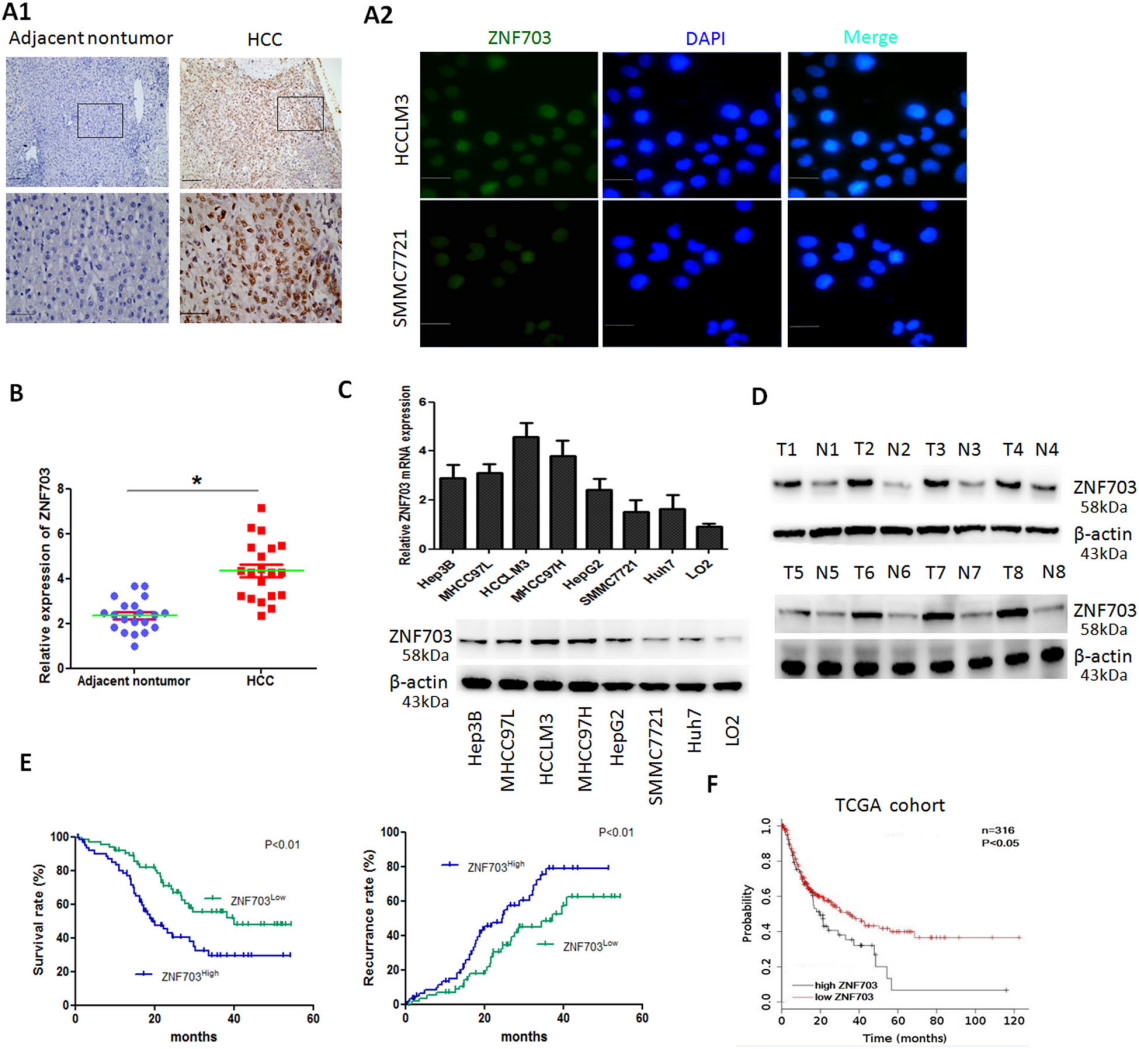
ZNF703 expression is upregulated in HCC and associated with poor prognosis. **A1** The immunohistochemical (IHC) staining of ZNF703 expressionin 128 pairs of HCC tissues and adjacent nontumor tissues Scale bars: 100 μm (low magnification) and 50 μm (high magnification); **A2** Immunofluorescentimages of HCCLM3 and SMMC7721 cells stained for ZNF703 (green) and 4′, 6-diamidino-2-phenylindole (DAPI; blue) Scale bars: 50 μm. **B** Real-time PCR analysis of ZNF703 mRNA expression in 20 pairs of HCC specimens (T) and adjacent nontumor tissues (N). **C** Real-time PCR and western blotting analysis of ZNF703 level in the LO2 cell line and seven HCC cell lines. **D** Representative protein expression of ZNF703 in HCC tissues (T) and adjacent nontumor tissues (N) were detected by western blotting analysis. **E** Kaplan–Meier analysis of the correlation between ZNF703 expression and overall survival or recurrence of 128 pairs HCC patients. **F** The prognostic value of ZNF703 was confirmed in TCGA cohort. The log-rank test was used to calculate *P*-values. *P < 0.05.

To explore the expression patterns of ZNF703 in HCC and its clinical significance, we first detected ZNF703 expression in 128 pairs of HCC tissues and adjacent normal tissues by using immunohistochemistry (IHC). The results showed that ZNF703 was primarily localized to the nucleus and was highly expressed in HCC tissues compared to adjacent nontumor tissues (Fig. 1A1). Further, we analyzed the relationship between clinicopathologic characteristics and ZNF703 expression levels in 128 HCC cases. High expressionof ZNF703 was significantly associated withmore tumor number, larger tumor size, poor tumor differentiation, tumor vascular invasion, loss of tumor encapsulation, morelymph node metastasisand advanced tumor-node metastasis (TNM) stage (Table 1). The ZNF703 protein immunostaining was mainly localized in the nucleus of HCC cells (Fig. 1A2). To further investigate these phenomena, we examined the expression of ZNF703 mRNAs and proteins in 20 HCC tumor specimens and the adjacent nontumor tissues, we found that ZNF703 mRNA and protein expressions were apparently higher in HCC samples compared with those in adjacent nontumor tissues (Fig. 1B, D). Moreover, we evaluated ZNF703 mRNA and protein expression in in various HCC cell lines and healthy liver cells by RT-qPCR and western blot. ZNF703 expressions were remarkably upregulated in HCC cell lines compared to immortalized liver cell line (L02) (Fig. 1C). In addition, ZNF703 overexpression was also associated with poor prognosis. The Kaplan–Meier analysis showed that patients with ZNF703 higher expression had shorter overall survival times and higher recurrence rates than those with ZNF703 lower expression (Fig. 1E). Multivariateanalyses indicated that ZNF703 overexpression as an independent predictor of overall survival (supplementary Table S3). The data from TCGA database determined the clinical implication of ZNF703 in HCC (Fig. 1F). These results demonstrated that ZNF703 is associated with poor tumor progression and could be served as an indicator for predicting the prognosis of patients with HCC.

**Table 1:** Correlation between expression of ZNF703 protein and clinicopathological characteristics in HCC patients (*n* = 128).

Variables		ZNF703		
	*n*	negative expression	positive expression	*P* Value
*Gender*				
Male	108	48	60	
Female	20	8	12	0.713
*Age (years)*				
≤50	54	24	30	
>50	74	32	42	0.892
*HBsAg*				
Positive	102	44	58	
Negative	26	12	14	0.782
*Serum AFP*				
≤20 ng/ml	28	11	17	
>20 ng/ml	100	45	55	0.590
*Cirrhosis*				
Yes	110	46	64	
No	18	10	8	0.276
*Differentiation*				
Well-moderate	98	48	50	
Poor-undifferentiated	30	8	22	**0.031**
*Tumor size*				
≤5 cm	27	7	20	
>5 cm	101	49	52	**0.036**
*Tumor number*				
Single	69	35	34	
Multiple	59	21	38	0.085
*Tumor encapsulation*				
Absent	72	35	37	
Present	56	21	35	0.209
*Vascular invasion*				
Yes	25	5	20	
No	103	51	52	**0.008**
*LNM*				
Yes	23	5	18	
No	105	51	54	**0.019**
*TNM*				
I~II	73	38	35	
III~IV	55	18	37	**0.029**

*AFP* alpha-fetoprotein, *LNM* lymph node metastasis, *HBsAg* hepatitis B surface antigen.

*P* values by Chi-square test.

Bold values indicate statistical significance, *P* < 0.05.

### ZNF703 enhances HCC cell invasion and metastasis

**Fig. 2:**
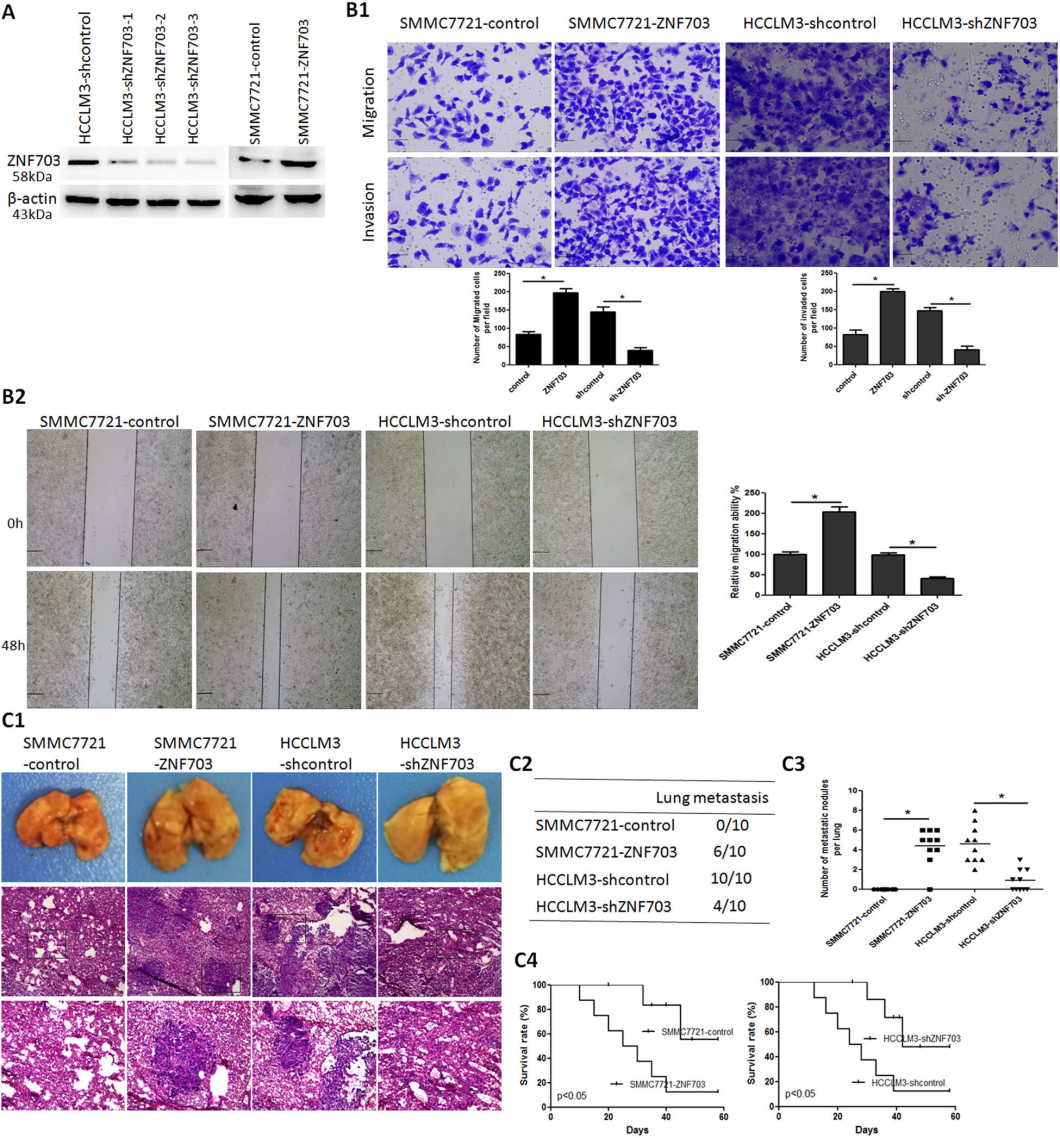
ZNF703 promotes hepatoma cell invasion and metastasis both in vitro and vivo. **A** Western blot analysis level of ZNF703 protein expression in the stable cell lines infected with LV-ZNF703 or LV-shZNF703-1,-2, or-3. **B** Themigration and invasion properties of SMMC7721-ZNF703 and HCCLM3-shZNF703 were detected by wound-healing (**B2**) and transwell assays (**B1**). Scale bars: 250 μm (wound-healing) and 75 μm (transwell). **C** In vivo metastasis assays, (**C1**) Lung representative morphology and H&E staining, (**C2**) incidence of lung metastasis, (**C3**) number of metastatic lung foci, and (**C4**) overall survival of nude mice in four groups (*n* = 10) are shown. Scale bars: 250 μm (low magnification) and 100 μm (high magnification). Data are presented as mean ± S.D for three independent experiments. *P < 0.05.

To evaluate the role of ZNF703 in HCC metastasis, we established two stable transfection cell lines, ZNF703 overexpression cell line SMMC7721-ZNF703 and knock down cell line HCCLM3-shZNF703. Western blot analysis was used to confirm the upregulation and knockdown of ZNF703 expressions (Fig. 2A). There are three target sites selected as knock-down candidates for ZNF703 expression. Of the three, target site three was selected for next study. Overexpression of ZNF703 markedly enhanced the migration and invasion activities of low metastatic SMMC7721 cells. Conversely, knockdown of ZNF703 significantly suppressed the migration and invasion potentials of high metastatic HCCLM3 cells (Fig. 2B1, B2). Thein vivo metastatic assay demonstrated that ZNF703 overexpression in SMMC7721 cells significantly enhanced the incidence of lung metastasis and the number of metastatic lung nodules compared with the control. However, ZNF703 knockdown in HCCLM3 cells significantly reduced the incidence of lung metastasis and the number of metastatic lung nodules compared with the control (Fig. 2C1, C2, C3). Consistent withthese results, downregulation of ZNF703 in HCCLM3 cells remarkably increased the overall survival time of xenografted mice compared with the control, whereas upregulation of ZNF703 in SMMC7721 cells dramatically decreased the overall survival time of xenografted mice compared to the control (Fig. 2C4). These data indicated that ZNF703 enhances HCC invasion and metastasis.

### ZNF703 induces EMT by transactivating CLDN4 expression in HCC Cells

**Fig. 3:**
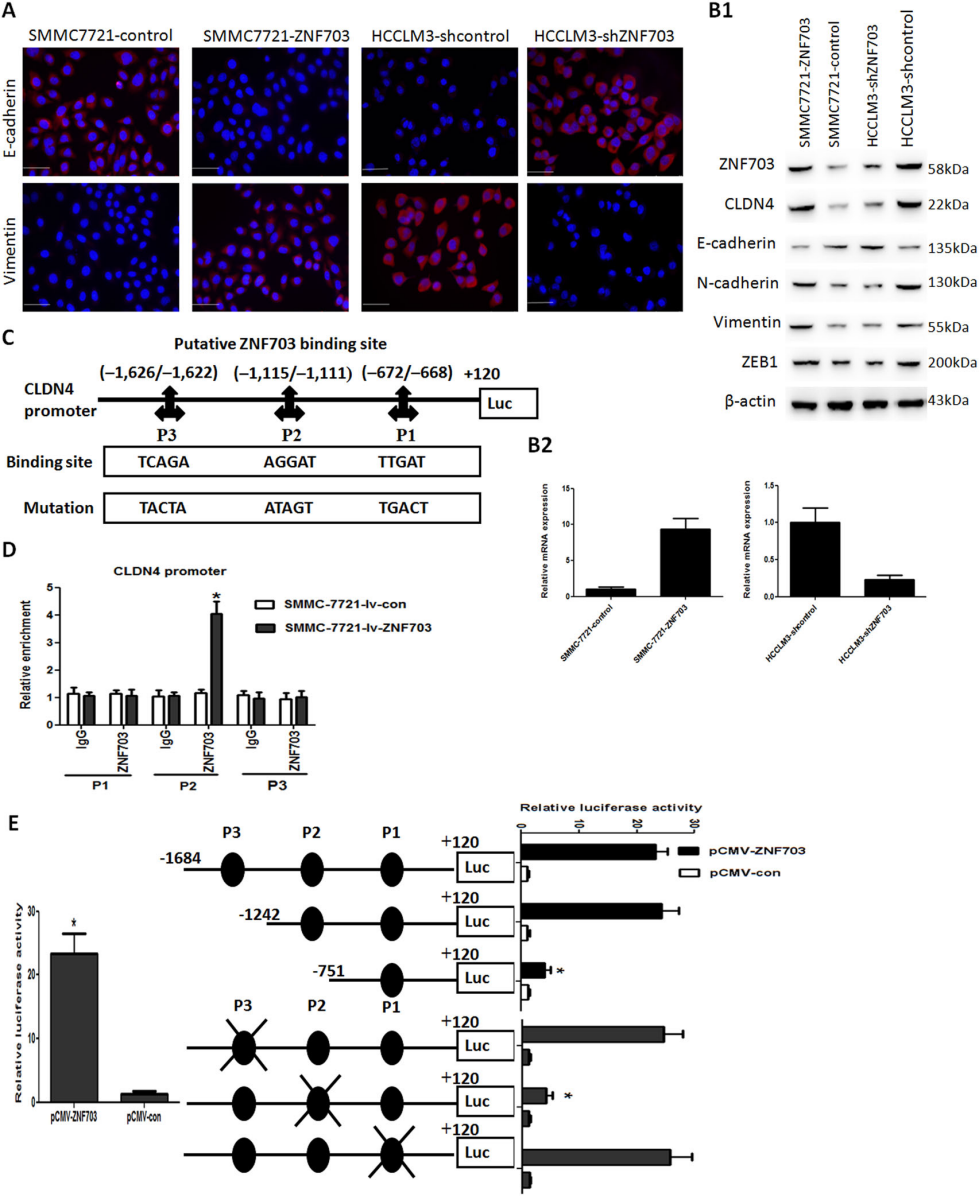
ZNF703 facilitates hepatoma cell metastasis by transactivating CLDN4 expression. **A** Immunofluorescence staining show that decreased expression of an epithelial marker (E-cadherin) and increased expression of a mesenchymal marker (vimentin) in SMMC7721-ZNF703 cell, whereas increased expression of an epithelial marker (E-cadherin) and decreased expression of a mesenchymal marker (vimentin) in HCCLM3-shZNF703 cell. Scale bars: 50 μm. **B1** Western blotting analysis of ZNF703, CLDN4, E-cadherin, N-cadherin, Vimentin, and ZEB1 expression in the SMMC7721-ZNF703 and HCCLM3-shZNF703 cells. **B2** Real-time PCR analysis of CLDN4 level in the SMMC7721-ZNF703 and HCCLM3-shZNF703 cells. **C** Prediction and validation of putative ZNF703-binding sites in the CLDN4 promoter and mutations in corresponding binding sites. **D** ChIP and real-time PCR assays demonstrate that ZNF703 binds directly to the CLDN4 promoter in SMMC7721-ZNF703 cells. **E** A luciferase reporter assay shows that ZNF703 apparently forces CLDN4 promoter activity. Deletionand selective mutagenesis are used to determine ZNF703-responsive regions in the CLDN4 promoter. Schematic representations of serially truncated andmutated CLDN4 promoters (left) and the corresponding relative luciferase activity (right) are shown. Data are presented as mean ± S.D for three independent experiments. *P < 0.05.

Mounting evidence has suggested that EMT plays important roles in HCC metastasis^22^. In our study, immunofluorescence (IF) indicated that overexpression of ZNF703 in SMMC7721 cellsreduced E-cadherin expression and forced vimentin expression compared with the control, whereas knockdown of ZNF703 in HCCLM3 cells enhanced E-cadherin expression and reduced vimentin expression compared with control group (Fig. 3A). Western blotting analysis indicated that overexpression of ZNF703 in SMMC7721 cells reduced expression of E-cadherin and upregulated expression of N-cadherin, Vimentin and ZEB1 compared to the control. In contrast, knockdown of ZNF703 in HCCLM3 cells enhanced E-cadherin expression and reduced expression of E-cadherin and reduced expression of N-cadherin, Vimentin, and ZEB1 compared to the control (Fig. 3B1). These results suggested that ZNF703 can induce EMT in HCC cells.

To further explore the molecular mechanism through which ZNF703 enhanced HCC cell metastasis and invasion, RNA sequencing were used to compare mRNA expression profiles between HCCLM3-shZNF703 and HCCLM3-control cells. Silencing ZNF703 changedthe expression of much genes (supplementary Table S2). Among these genes, CLDN4 was remarkably decreased by downregulation of ZNF703 in HCCLM3 cells, which attracted our attention, because it is involved in the metastasis of many cancers, such as gastric cancer^12,23^, ovarian cancer^14,19^, prostate cancer^15^, breast cancer^16^ and pancreatic cancer^13^. Real-time PCR and western blotting analysis showed that overexpression of ZNF703 in SMMC7721 cells increased the expression of CLDN4 compared to the control. In contrast, knockdown of ZNF703 in HCCLM3 cells reduced expression of CLDN4 compared to the control (Fig. 3B1, B2). To determine whether CLDN4 is the direct target of ZNF703, we analyzed the promoter sequence of CLDN4 and identified three putative ZNF703-binding sites by using UCSC and TFSEARCH bioinformatics softwares (Fig. 3C). Luciferase reporter assay showed that ZNF703 transactivated CLDN4 promoter activity. Serial deletion and site-specific mutagenesis showed that the second ZNF703-binding site of the CLDN4 promoterwas critical for ZNF703-induced CLDN4 transactivation (Fig. 3E). A chromatin immunoprecipitation (ChIP) assay further confirmed ZNF703 protein was enriched to the second ZNF703-binding site in the CLDN4 promoter in HCC cells (Fig. 3D). These results suggested that CLDN4 is the direct target ofZNF703.

### CLDN4 overexpression promotes HCC invasion and metastasis

**Fig. 4:**
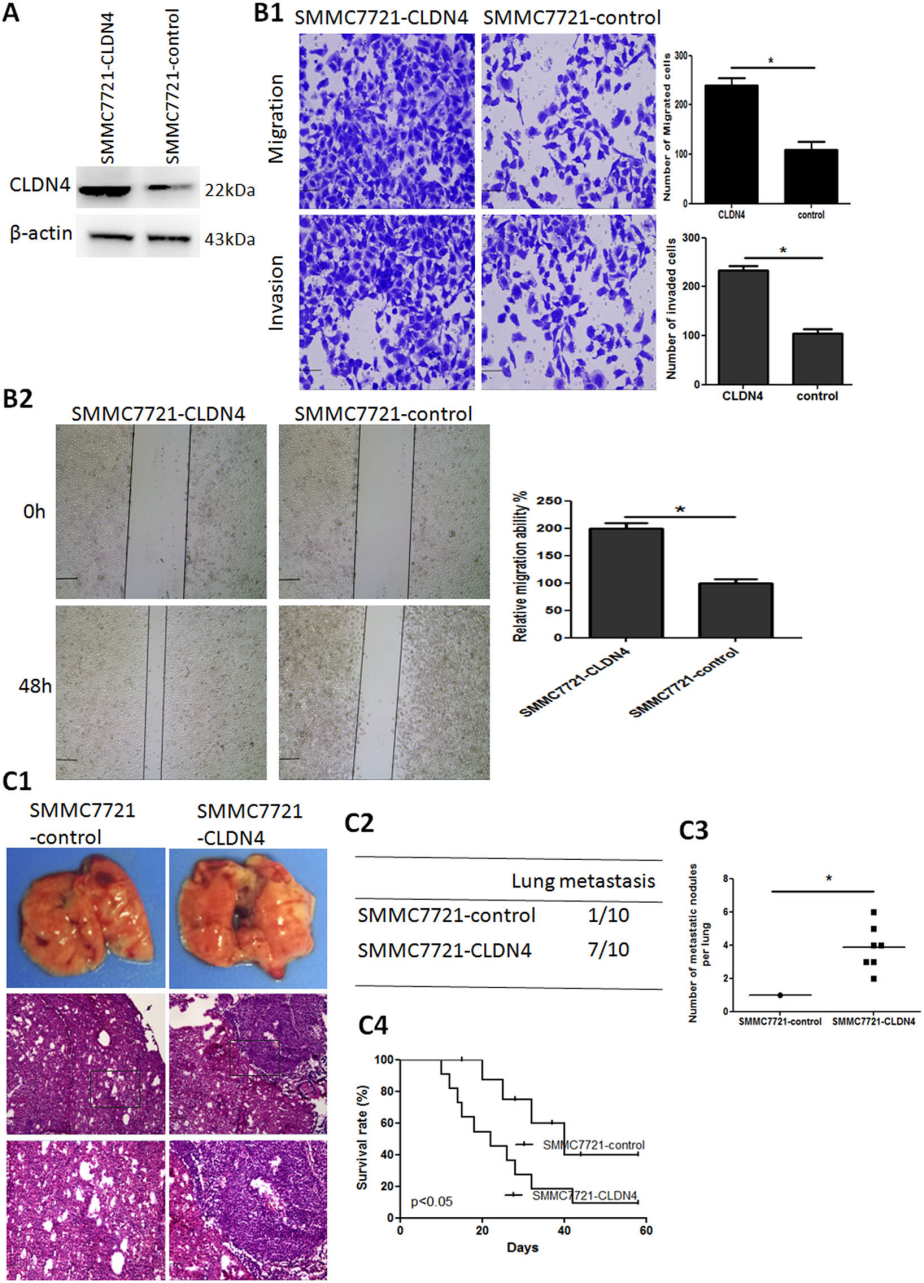
CLDN4 overexpression enhances HCC invasion and metastasis. **A** Upregulation of CLDN4 expression in SMMC7721-CLDN4 cell is assessed by Western blotting. **B** Transwell (**B1**) and wound-healing (**B2**) assay analysis of migration and invasion properties of SMMC7721-CLDN4 and SMMC7721-control cells. Scale bars: 250 μm (wound-healing) and 75 μm (transwell). **C** In vivo metastasis assays, (**C1**) Lung representative morphology and H&E staining, (**C2**) incidence of lung metastasis, (**C3**) number of metastatic lung foci, and (**C4**) overall survival of nude mice in SMMC7721-CLDN4 and SMMC7721-control groups (*n* = 10) are shown. Scale bars: 250 μm (low magnification) and 100 μm (high magnification). Data are presented asmean±S.D for three independent experiments. *P < 0.05.

To investigate the function of CLDN4 in HCC cells, SMMC7721 cells were transduced with Lv-CLDN4 lentivirus. Western blotting analysis showed that CLDN4 expression was upregulated compared with the control (Fig. 4A). Transwell and wound-healing assays revealed thatoverexpression of CLDN4 markedly enhanced the migration and invasion activities of SMMC7721 cells (Fig. 4B1, B2). The in vivo metastatic assay demonstrated that the incidence of lung metastases and the number of metastatic lung nodules in mice implanted with SMMC7721-CLDN4 was higher than those with SMMC7721-control (Fig. 4C1, C2, C3). Consistent with these results, upregulation of CLDN4 in SMMC7721 cells dramatically decreased the overall survival rate of xenografted mice compared to the control (Fig. 4C4).

### CLDN4 is essential for ZNF703-mediated HCC invasion and metastasis

**Fig. 5:**
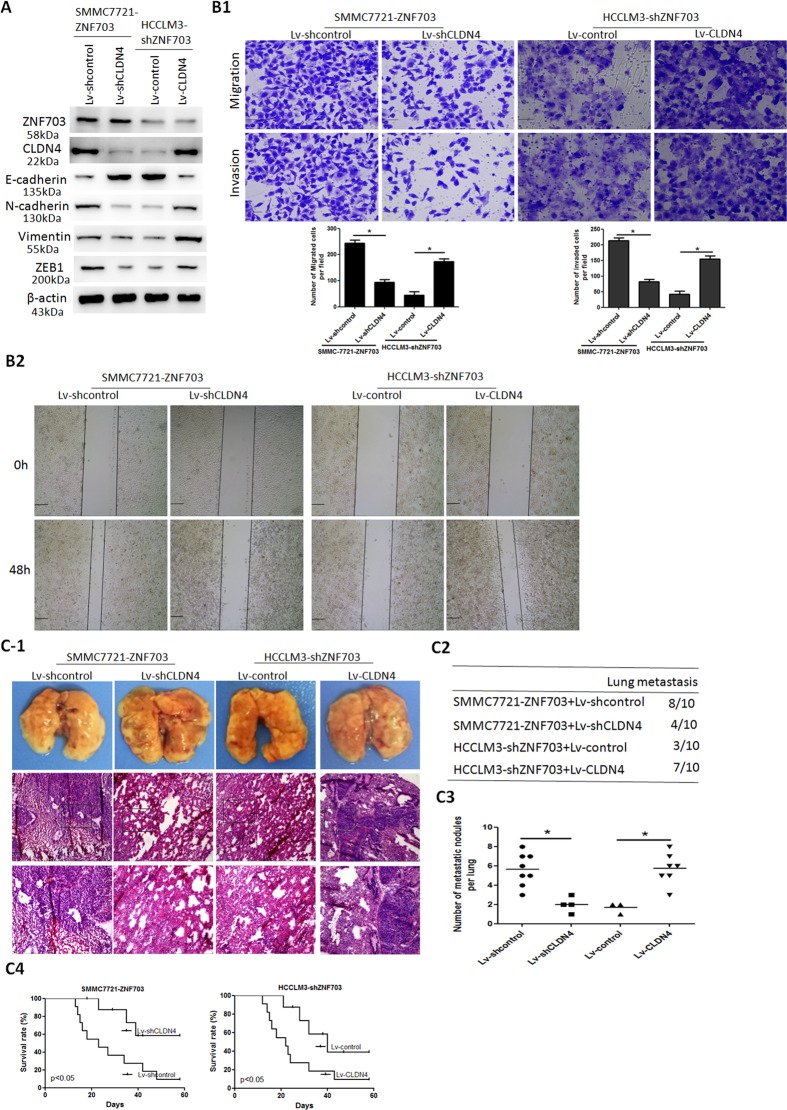
CLDN4 is essential for ZNF703-mediated HCC invasion and metastasis. **A** Western blotting analysis of ZNF703, CLDN4, E-cadherin, N-cadherin, Vimentin, and ZEB1 expression in upregulating or downregulating CLDN4 in the SMMC7721-ZNF703 or HCCLM3-shZNF703 cells, respectively. **B** Transwell (**B1**) and wound-healing (**B2**) assays demonstrate that upregulation of CLDN4 increases migration and invasion abilities of HCCLM3-shZNF703 cells, whereas downregulation of CLDN4 decreases migration and invasion abilities of SMMC7721-ZNF703 cells. Scale bars: 250 μm (wound-healing) and 75 μm (transwell). **C** In vivo metastasis assays, (**C1**) Lung representative morphology and H&E staining, (**C2**) incidence of lung metastasis, (**C3**) number of metastatic lung foci, and (**C4**) overall survival of nude mice in SMMC7721-ZNF703 cells downregulating CLDN4 and HCCLM3-shZNF703 cells upregulating CLDN4 groups (*n* = 10) are shown. Scale bars: 250 μm (low magnification) and 100 μm (high magnification). Data are presented as mean ± S.D for three independent experiments. *P < 0.05.

To validate whether CLDN4 is involved in ZNF703-mediated HCC invasion and metastasis, we established two stable cell lines: upregulation of CLDN4 expression in HCCLM3-shZNF703 cells and downregulation of CLDN4 expression in SMMC7721-ZNF703 cells. Previous studies showed that CLDN4 can enhance cell invasion and metastasis by promoting EMT^18,20^. Our data showed that ZNF703 increased CLDN4 expression and therefore induced EMT. We wondered whether upregulated EMT which caused by ZNF703-overexpression could be reversed by the reduction of CLDN4 expression. To explore this we performed western blot analysis, results confirmed that high levels of E-cadherin and low levels of vimentin, N-cadherin and ZEB1 were observed in the SMMC7721-ZNF703 cells downregulating CLDN4, whereas the opposite results were observed in the HCCLM3-shZNF703 cells with upregulated CLDN4 expression (Fig. 5A). Overexpression of CLDN4 restored the decreased migration and invasion potentials induced by the silencing of ZNF703, whereas the knockdown of CLDN4 attenuated ZNF703-promoted migration and invasion potentials (Fig. 5B1, B2). The in vivo metastatic assay showed that CLDN4 overexpression in HCCLM3-shZNF703 cells significantly enhanced the incidence of lung metastasis and the number of metastatic lung nodules and shortened the overall survival time of xenografted mice compared with the control. However, CLDN4 knockdown in SMMC7721-ZNF703 cells significantly reduced the incidence of lung metastasis and the number of metastatic lung nodules and extended the overall survival time of xenografted mice compared with the control (Fig. 5C1, C2, C3, C4).

**Fig. 6:**
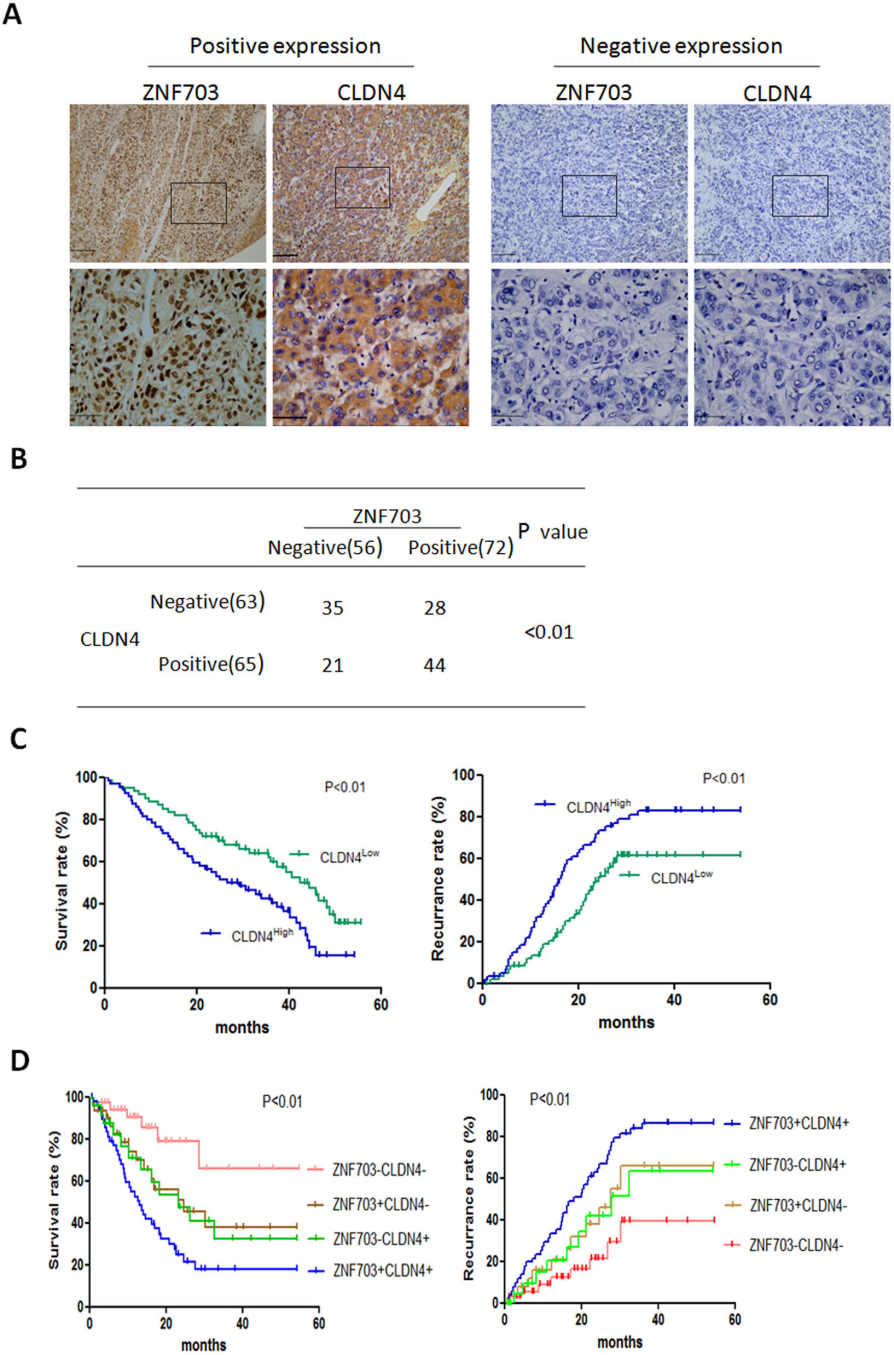
ZNF703 was positively correlated with CLDN4 expression in HCC tissues. **A** Immunohistochemistry staining analysis of ZNF703 and CLDN4 expression in HCC tissues and adjacent nontumor tissues. Scale bars: 100 μm (low magnification) and 50 μm (high magnification). **B** The correlation between the expression of ZNF703 and CLDN4 in HCC tissues. **C** Kaplan–Meier analysis of the correlation between CLDN4 expression and overall survival or recurrence of 128 pairs HCC patients. **D** Correlation of ZNF703/CLDN4 coexpression with overall survival and recurrence in HCC patients. The log-rank test was used to calculate *P*-values.

IHC results showed that CLDN4 was primarily localized within cytoplasm and was highly expressed in HCC tissues compared to adjacent nontumor tissues (Fig. 6A). ZNF703 was positively correlated with CLDN4 expressionin HCC tissues (Fig. 6B). CLDN4 overexpression was apparently associated with poor prognosis. Patients with CLDN4 higher expression had shorter overall survival times and higher recurrence rates than those with CLDN4 lower expressions (Fig. 6C). These results suggested thatCLDN4 overexpression promoted HCC metastasis and was associated with poor prognosis. Kaplan-Meier’s analysis showed the expression pattern of ZNF703(+) CLDN4(+) was correlated with the lowest overall survival times and the highest recurrence (Fig. 6D). These results suggested that ZNF703 induce EMT by transactivating CLDN4 expression in HCC Cells.

### ZNF703 expression in HCC is related to drug susceptibility of sorafenib

**Fig. 7:**
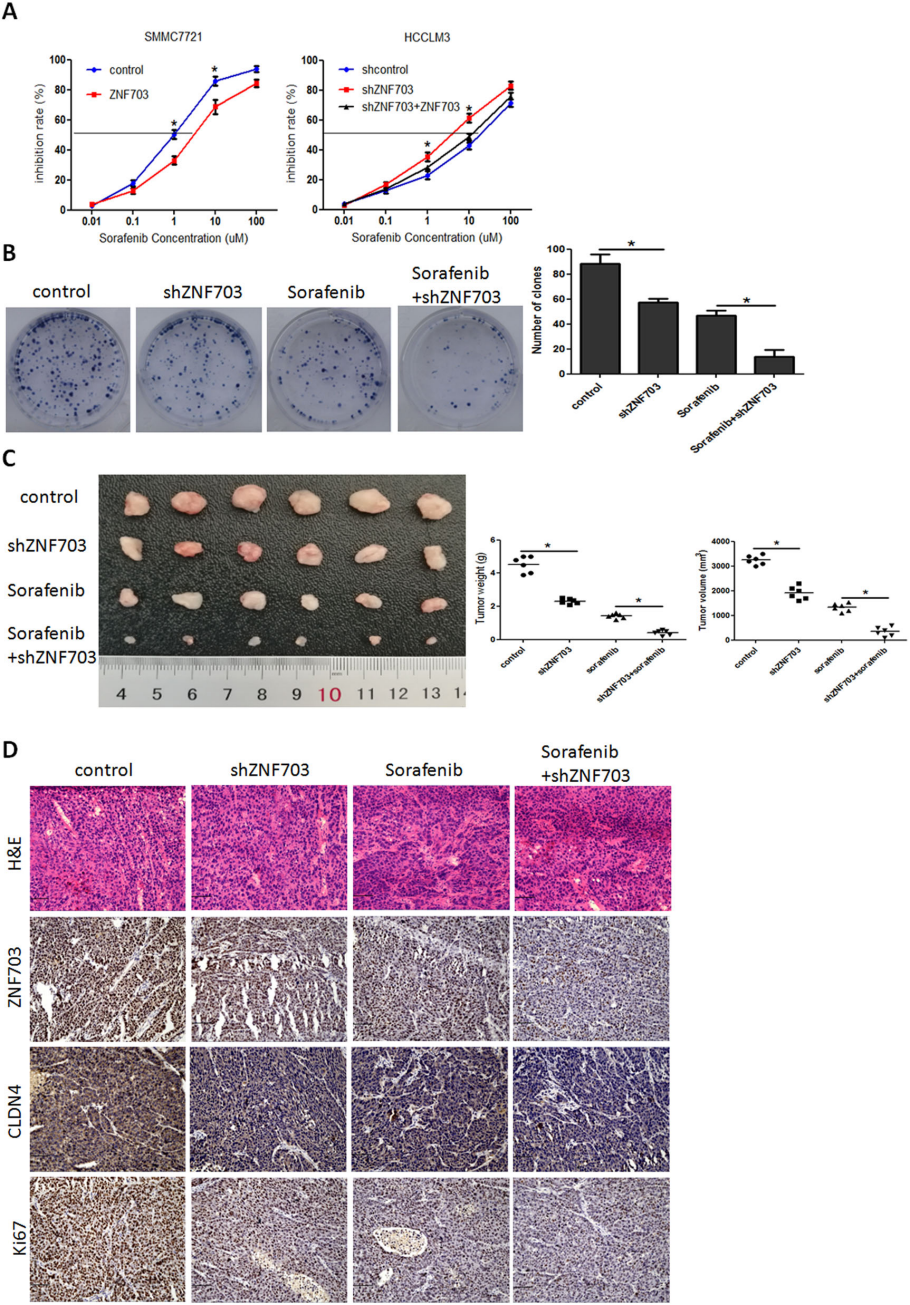
Depletion of ZNF703 enhances the sensitivity of HCC cells to sorafenib treatment. **A** Overexpression of ZNF703 in SMMC7721 decreased their sensitivity to sorafenib treatment, whereas depletion of ZNF703 in HCCLM3 cells increased their sensitivity to sorafenib treatment, which be rescued by ZNF703 overexpression. **B** The influence of silencing ZNF703 or treatment with sorafenib on the effects of colony formation in HCCLM3 cell. **C** The HCCLM3-shZNF703 or corresponding control cells were subcutaneously inoculated into nude mice. After subcutaneous inoculation, mice were given sorafenib (2 mg/kg) per 2 days. Then, 4 weeks after giving sorafenib, mice were harvested and xerographs were collected. Comparison of tumor weights and tumor volumes for each groups (*n* = 6). **D** Expression of ZNF703, CLDN4 and Ki67 was evaluated by immunohistochemistry in xenografts derived from HCC cells. Scale bars: 75 μm. Data are presented as mean±S.D for three independent experiments. *P < 0.05.

Recent studies reported that EMT is involved in HCC resistance tosorafenib^24–26^. We explored the role of ZNF703 in HCC sensitivity to sorafenib in this study. The sorafenib IC50 value of HCCLM3-shZNF703 cells was decreased remarkably compared to the shcontrol, whereas the sorafenib IC50 value of SMMC7721-ZNF703 increased markedly compared with the control (Fig. 7A). The colony formation assay revealed that Sorafenib administration significantly reduced HCCLM3 cells’proliferation. Knockdown of ZNF703 enhanced the effect of Sorafenib on MHCC97-H cells’proliferation (Fig. 7B). Furthermore, the in vivo antitumor assay demonstrated that tumor load in both control+sorafenib and shZNF703+sorafenib groups were significantly decreased compared to those in control and shZNF703 groups, which indicated that ZNF703 depletion increased the effect of Sorafenib on subcutaneous tumor growth (Fig. 7C). Consistent with in vivo results, immunohistochemistry of xenografts generated from control+sorafenib and shZNF703+sorafenib cells revealed an obvious reduction in ZNF703, CLDN4 and Ki67 expression (Fig. 7D).

## Discussion

Zinc Finger Protein 703 (ZNF703) has been reported to be abnormally expressed in many different malignancies and its aberrant expression is involved in tumor progression^27–30^. However, there are few reports on the expression and function of ZNF703 in HCC. In this study, we demonstrated that ZNF703 were highly expressed in human HCC tissues compared to adjacent nontumor tissues. In addition, HCC patients with ZNF703 higher expression levels had shorter overall survival times and higher recurrence rates than those with lower ZNF703 expressions. These clinical studies suggested thatZNF703 may conduce to HCC malignant progression.

EMT has an important role in HCC migration and invasion^31,32^. In tumor EMT process, the expression of epithelial markers E-cadherin and zonula occluden-1 were downregulated, while the expression of mesenchymal markers vimentin and N-cadherin were upregulated^33^. In this study, we found that the overexpression of ZNF703 increased expression of mesenchymal markers (N-cadherin and vimentin) and decreased expression of epithelial marker (E-cadherin), whereas the knockdown of ZNF703 increased expression of epithelial marker (E-cadherin) and decreased expression of mesenchymal markers (N-cadherin and vimentin). Thus, HCC cells that with upregulated ZNF703 levels become more aggressive because the activation of EMT. Furthermore, RNA sequencing was used to compare mRNA expression profiles between HCCLM3-shZNF703 and HCCLM3-control cells. We found that Silencing of ZNF703 changed the expression of multiple genes. Among these genes, CLDN4 was remarkably decreased which caused by the downregulation of ZNF703 in HCCLM3 cells. Overexpression of CLDN4 promotes proliferation, invasion, and EMT and is associated with poor prognosis in various types of human cancers^18,19^. A previous study reported that CLDN4 overexpression may promote ovarian tumorigenesis and metastasis through the activation of MMP2^19^. Consistently, overexpression of CLDN4 enhanced EMT in the ovarian cancer cells via a pathway involving PI3K/Akt and EMT transcription factor Twist1^18^. These earlier studies suggested that CLDN4 plays an important role in cancer metastasis. However, the biological function of CLDN4 in HCC metastasis remains unknown. In this study, we observed that CLDN4 overexpression promoted HCC metastasis and was associated with poor prognosis. Using serial deletion site-specific mutagenesis and chromatin immunoprecipitation (ChIP), we showed that CLDN4 was a direct target of ZNF703. Overexpression of CLDN4 restored the decreased migration and invasion potentials induced by silencing ZNF703, whereas the knockdown of CLDN4 attenuated ZNF703 promoted migration and invasion potentials. In addition, ZNF703 was positively correlated with CLDN4 expression and the coexpression of these two genes was correlated with the lowest overall survival times and the highest recurrence in human HCC patients. Thus, ZNF703 promoted HCC metastasis and induced EMT by transactivating CLDN4 expression in HCC Cells.

Several recent studies have reported that EMT contributes to HCC resistance to sorafenib^24,26^. A recent study reported that Gal-1 overexpression activated the FAK/PI3K/AKT pathway by upregulating expression of αvβ3 integrin, leading to enhanced HCC invasion via EMT and sorafenib resistance^24^. Consistently, cryab overexpression upregulates ERK phosphorylation by complexing with 14-3-3f, leading to an increase in HCC invasion through EMT and resistance to sorafenib^26^. In this study, we observed that the overexpression of ZNF703 resulted in HCC sorafenib resistance both in vitro and in vivo.

In conclusion, we describeda new biological function of ZNF703 in HCC metastasis. ZNF703 overexpression promotes HCC metastasis and sorafenib resistance by modulating EMT via upregulating CLDN4. Thus, ZNF703 may be a potential target for new therapies and a candidate prognostic biomarker for predicting drug susceptibility of sorafenib.

## Supplementary information

Supplementary Table S1

Supplementary Table S2

Supplementary Table S3
