# Supplementary material for: Zinc finger protein 703 induces EMT and sorafenib resistance in hepatocellular carcinoma by transactivating CLDN4 expression
Source: Cell Death Dis. 2020 Apr 8;11(4):225. doi: 10.1038/s41419-020-2422-3 (PMC7142083; doi:10.1038/s41419-020-2422-3)
Supplement: Supplementary file 1 — Supplementary Table S1 [file 41419_2020_2422_MOESM1_ESM.docx]

**Supplementary Table S1 Primer sequences used in the study**

| Primer name | Primer sequences | Enzyme |
| --- | --- | --- |
| Primers for CLDN4 promoter construct: | |  |
| (-1684/+120) CLDN4 sense: | 5’-TATAGGTACCCTCAGCTCACAGAGATCT-3’ | KpnI |
| (-1242/+120) CLDN4: | 5’-TATAGGTACCACAGCGACATTCCGTCTC-3’ | KpnI |
| (-751/+120) CLDN4: | 5’-TATAGGTACCCTCAGAGATGGTTCCTGG-3’ | KpnI |
| Antisense: | 5’-ATATAAGCTTAAACACAGTGGTCCTGCC-3’ | HindIII |
| Primers for CLDN4 promoter site-directed mutagenesis: | |  |
| binding site 1 mutation sense: | 5’-GCCCTGGGGCtactaTGCCAGCGGGA -3’ |  |
| binding site 1 mutation antisense: | 5’- TCCCGCTGGCAtagtaGCCCCAGGGC-3’ |  |
| binding site 2 mutation sense: | 5’-CTGTTATGATCatagtCCGAACTGCG -3’ |  |
| binding site 2 mutation antisense: | 5’-CGCAGTTCGGactatGATCATAACAG -3’ |  |
| binding site 3 mutation sense: | 5’-GGTTTTTATCCtgactAGAAACCAGC -3’ |  |
| binding site 3 mutation antisense: | 5’-GCTGGTTTCTagtcaGGATAAAAACC -3’ |  |
| Primers used for ChIP in the CLDN4 promoter: | |  |
| binding site 1 sense: | 5’-GTTCCCAGAAGACGTGGA-3’ |  |
| binding site 1 antisense: | 5’-GGATTGCAGGCGTGAGCT-3’ |  |
| binding site 2 sense: | 5’-ACCACTGCACTATAGCCT-3’ |  |
| binding site 2 antisense: | 5-CAGTCTGAGATACACATG-3’ |  |
| binding site 3 sense: | 5’-CATCTCATTTCACGTCTG-3’ |  |
| binding site 3 antisense: | 5’-CCAGGCTGGTCTCAAACT-3’ |  |
|  |  |  |
