# Supplementary material for: Zinc finger protein 703 induces EMT and sorafenib resistance in hepatocellular carcinoma by transactivating CLDN4 expression
Source: Cell Death Dis. 2020 Apr 8;11(4):225. doi: 10.1038/s41419-020-2422-3 (PMC7142083; doi:10.1038/s41419-020-2422-3)
Supplement: Supplementary file 2 — Supplementary Table S2 [file 41419_2020_2422_MOESM2_ESM.docx]

**Supplementary Table S2 List of genes downregulated in HCCLM3-shZNF703 versus HCCLM3-shcontrol cells**

| Gene description log2FoldChange   \| C15orf38-AP3S2 \| C15orf38-AP3S2 readthrough [Source:HGNC Symbol;Acc:HGNC:38824] \| -9.30399 \| \| --- \| --- \| --- \| \| AMIGO3 \| adhesion molecule with Ig like domain 3 [Source:HGNC Symbol;Acc:HGNC:24075] \| -7.0631 \| \| AC008764.1 \| novel transcript \| -6.35381 \| \| TOMM6 \| translocase of outer mitochondrial membrane 6 [Source:HGNC Symbol;Acc:HGNC:34528] \| -6.11203 \| \| AC026954.2 \| novel transcript \| -5.56857 \| \| TMEM256-PLSCR3 \| TMEM256-PLSCR3 readthrough (NMD candidate) [Source:HGNC Symbol;Acc:HGNC:49186] \| -5.42853 \| \| CORO7-PAM16 \| CORO7-PAM16 readthrough [Source:HGNC Symbol;Acc:HGNC:44424] \| -5.02844 \| \| CACNG8 \| calcium voltage-gated channel auxiliary subunit gamma 8 [Source:HGNC Symbol;Acc:HGNC:13628] \| -4.55781 \| \| ANO7 \| anoctamin 7 [Source:HGNC Symbol;Acc:HGNC:31677] \| -4.26142 \| \| MYC \| MYC proto-oncogene, bHLH transcription factor [Source:HGNC Symbol;Acc:HGNC:7553] \| -4.22385 \| \| MT-TC \| mitochondrially encoded tRNA cysteine [Source:HGNC Symbol;Acc:HGNC:7477] \| -4.18389 \| \| AL591684.2 \| novel transcript \| -4.07935 \| \| AC074386.1 \| novel transcript, antisense to OR2A1 and ARHGEF5 \| -3.96746 \| \| AC079781.5 \| novel transcript \| -3.88524 \| \| ANP32AP1 \| acidic nuclear phosphoprotein 32 family member A pseudogene 1 [Source:HGNC Symbol;Acc:HGNC:42949] \| -3.84633 \| \| LINC00598 \| long intergenic non-protein coding RNA 598 [Source:HGNC Symbol;Acc:HGNC:42770] \| -3.71444 \| \| CLDN4 \| claudin 4 [Source:HGNC Symbol;Acc:HGNC:2046] \| -3.68567 \| \| AC006213.2 \| uncharacterized LOC100505715 [Source:NCBI gene;Acc:100505715] \| -3.67648 \| \| PRR29 \| proline rich 29 [Source:HGNC Symbol;Acc:HGNC:25673] \| -3.53679 \| \| UGT1A5 \| UDP glucuronosyltransferase family 1 member A5 [Source:HGNC Symbol;Acc:HGNC:12537] \| -3.48219 \| \| SCN2A \| sodium voltage-gated channel alpha subunit 2 [Source:HGNC Symbol;Acc:HGNC:10588] \| -3.4727 \| \| INS-IGF2 \| INS-IGF2 readthrough [Source:HGNC Symbol;Acc:HGNC:33527] \| -3.42851 \| \| AC113189.4 \| novel transcript \| -3.33515 \| \| AP000275.2 \| novel protein \| -3.31931 \| \| AP000866.6 \| novel transcript, sense intronic to MSANTD2 \| -3.29865 \| \| SPNS2 \| sphingolipid transporter 2 [Source:HGNC Symbol;Acc:HGNC:26992] \| -3.2612 \| \| ANKRD10-IT1 \| ANKRD10 intronic transcript 1 [Source:HGNC Symbol;Acc:HGNC:39891] \| -3.2612 \| \| SACS \| sacsin molecular chaperone [Source:HGNC Symbol;Acc:HGNC:10519] \| -3.24169 \| \| HSP90AB2P \| heat shock protein 90 alpha family class B member 2, pseudogene [Source:HGNC Symbol;Acc:HGNC:32537] \| -3.22272 \| \| RNF112 \| ring finger protein 112 [Source:HGNC Symbol;Acc:HGNC:12968] \| -3.18317 \| \| BCL2L2 \| BCL2 like 2 [Source:HGNC Symbol;Acc:HGNC:995] \| -3.06677 \| \| DUOXA2 \| dual oxidase maturation factor 2 [Source:HGNC Symbol;Acc:HGNC:32698] \| -3.03339 \| \| PSAT1P3 \| phosphoserine aminotransferase 1 pseudogene 3 [Source:HGNC Symbol;Acc:HGNC:43989] \| -3.01349 \| \| TACR2 \| tachykinin receptor 2 [Source:HGNC Symbol;Acc:HGNC:11527] \| -3.01328 \| \| CASP1 \| caspase 1 [Source:HGNC Symbol;Acc:HGNC:1499] \| -3.01328 \| \| LUZP2 \| leucine zipper protein 2 [Source:HGNC Symbol;Acc:HGNC:23206] \| -3.01328 \| \| AC079416.1 \| thyroid hormone receptor interactor 13 (TRIP13) pseudogene \| -3.01328 \| \| GCNT1 \| glucosaminyl (N-acetyl) transferase 1, core 2 [Source:HGNC Symbol;Acc:HGNC:4203] \| -2.98736 \| \| TMEM164 \| transmembrane protein 164 [Source:HGNC Symbol;Acc:HGNC:26217] \| -2.91595 \| \| PCDHA1 \| protocadherin alpha 1 [Source:HGNC Symbol;Acc:HGNC:8663] \| -2.88798 \| \| ZBED3-AS1 \| ZBED3 antisense RNA 1 [Source:HGNC Symbol;Acc:HGNC:44188] \| -2.88798 \| \| LRRC8E \| leucine rich repeat containing 8 VRAC subunit E [Source:HGNC Symbol;Acc:HGNC:26272] \| -2.87691 \| \| AC008074.2 \| uncharacterized LOC101927402 [Source:NCBI gene;Acc:101927402] \| -2.75043 \| \| YOD1 \| YOD1 deubiquitinase [Source:HGNC Symbol;Acc:HGNC:25035] \| -2.65216 \| \| SSTR5 \| somatostatin receptor 5 [Source:HGNC Symbol;Acc:HGNC:11334] \| -2.62815 \| \| AK4 \| adenylate kinase 4 [Source:HGNC Symbol;Acc:HGNC:363] \| -2.61913 \| \| LINC00342 \| long intergenic non-protein coding RNA 342 [Source:HGNC Symbol;Acc:HGNC:42470] \| -2.6161 \| \| ZNF445 \| zinc finger protein 445 [Source:HGNC Symbol;Acc:HGNC:21018] \| -2.61387 \| \| AL096870.2 \| novel transcript \| -2.60838 \| \| CLYBL \| citrate lyase beta like [Source:HGNC Symbol;Acc:HGNC:18355] \| -2.59845 \| \| PAEP \| progestagen associated endometrial protein [Source:HGNC Symbol;Acc:HGNC:8573] \| -2.59845 \| \| DPT \| dermatopontin [Source:HGNC Symbol;Acc:HGNC:3011] \| -2.59845 \| \| BTBD8 \| BTB domain containing 8 [Source:HGNC Symbol;Acc:HGNC:21019] \| -2.59841 \| \| CFAP161 \| cilia and flagella associated protein 161 [Source:HGNC Symbol;Acc:HGNC:26782] \| -2.59821 \| \| SSPO \| SCO-spondin [Source:HGNC Symbol;Acc:HGNC:21998] \| -2.59821 \| \| AC090515.4 \| novel transcript, antisense to FAM63B \| -2.59821 \| \| SPN \| sialophorin [Source:HGNC Symbol;Acc:HGNC:11249] \| -2.59821 \| \| MRPL34 \| mitochondrial ribosomal protein L34 [Source:HGNC Symbol;Acc:HGNC:14488] \| -2.5811 \| \| TM4SF4 \| transmembrane 4 L six family member 4 [Source:HGNC Symbol;Acc:HGNC:11856] \| -2.56357 \| \| C6orf223 \| chromosome 6 open reading frame 223 [Source:HGNC Symbol;Acc:HGNC:28692] \| -2.516 \| \| YPEL2 \| yippee like 2 [Source:HGNC Symbol;Acc:HGNC:18326] \| -2.51592 \| \| C7orf55-LUC7L2 \| C7orf55-LUC7L2 readthrough [Source:HGNC Symbol;Acc:HGNC:44671] \| -2.50646 \| \| BAAT \| bile acid-CoA:amino acid N-acyltransferase [Source:HGNC Symbol;Acc:HGNC:932] \| -2.49374 \| \| ZRSR2P1 \| ZRSR2 pseudogene 1 [Source:HGNC Symbol;Acc:HGNC:12456] \| -2.47292 \| \| SH3BP1 \| SH3 domain binding protein 1 [Source:HGNC Symbol;Acc:HGNC:10824] \| -2.45265 \| \| RNF6 \| ring finger protein 6 [Source:HGNC Symbol;Acc:HGNC:10069] \| -2.44876 \| \| STAG3 \| stromal antigen 3 [Source:HGNC Symbol;Acc:HGNC:11356] \| -2.44635 \| \| LDLRAD4 \| low density lipoprotein receptor class A domain containing 4 [Source:HGNC Symbol;Acc:HGNC:1224] \| -2.44635 \| \| ADM2 \| adrenomedullin 2 [Source:HGNC Symbol;Acc:HGNC:28898] \| -2.42532 \| \| TENT5A \| terminal nucleotidyltransferase 5A [Source:HGNC Symbol;Acc:HGNC:18345] \| -2.41413 \| \| PAPPA2 \| pappalysin 2 [Source:HGNC Symbol;Acc:HGNC:14615] \| -2.40575 \| \| DHDH \| dihydrodiol dehydrogenase [Source:HGNC Symbol;Acc:HGNC:17887] \| -2.40557 \| \| SSTR5-AS1 \| SSTR5 antisense RNA 1 [Source:HGNC Symbol;Acc:HGNC:26502] \| -2.34273 \| \| NID2 \| nidogen 2 [Source:HGNC Symbol;Acc:HGNC:13389] \| -2.33534 \| \| EMID1 \| EMI domain containing 1 [Source:HGNC Symbol;Acc:HGNC:18036] \| -2.33534 \| \| DOCK8 \| dedicator of cytokinesis 8 [Source:HGNC Symbol;Acc:HGNC:19191] \| -2.33515 \| \| TTN \| titin [Source:HGNC Symbol;Acc:HGNC:12403] \| -2.32449 \| \| ZNF324B \| zinc finger protein 324B [Source:HGNC Symbol;Acc:HGNC:33107] \| -2.32376 \| \| MRAS \| muscle RAS oncogene homolog [Source:HGNC Symbol;Acc:HGNC:7227] \| -2.31823 \| \| PAX5 \| paired box 5 [Source:HGNC Symbol;Acc:HGNC:8619] \| -2.28649 \| \| SERPINA3 \| serpin family A member 3 [Source:HGNC Symbol;Acc:HGNC:16] \| -2.27643 \| \| NRTN \| neurturin [Source:HGNC Symbol;Acc:HGNC:8007] \| -2.25039 \| \| LIPE \| lipase E, hormone sensitive type [Source:HGNC Symbol;Acc:HGNC:6621] \| -2.23588 \| \| TIGD1 \| tigger transposable element derived 1 [Source:HGNC Symbol;Acc:HGNC:14523] \| -2.20983 \| \| MYBPC1 \| myosin binding protein C, slow type [Source:HGNC Symbol;Acc:HGNC:7549] \| -2.20598 \| \| PCDHGA5 \| protocadherin gamma subfamily A, 5 [Source:HGNC Symbol;Acc:HGNC:8703] \| -2.20598 \| \| RTEL1 \| regulator of telomere elongation helicase 1 [Source:HGNC Symbol;Acc:HGNC:15888] \| -2.18834 \| \| HIST2H3A \| histone cluster 2 H3 family member a [Source:HGNC Symbol;Acc:HGNC:20505] \| -2.18341 \| \| GLI1 \| GLI family zinc finger 1 [Source:HGNC Symbol;Acc:HGNC:4317] \| -2.18341 \| \| PALM2 \| paralemmin 2 [Source:HGNC Symbol;Acc:HGNC:15845] \| -2.18329 \| \| GFAP \| glial fibrillary acidic protein [Source:HGNC Symbol;Acc:HGNC:4235] \| -2.12895 \| \| INHBE \| inhibin subunit beta E [Source:HGNC Symbol;Acc:HGNC:24029] \| -2.11774 \| \| HPN \| hepsin [Source:HGNC Symbol;Acc:HGNC:5155] \| -2.11289 \| \| SERPINB11 \| serpin family B member 11 (gene/pseudogene) [Source:HGNC Symbol;Acc:HGNC:14221] \| -2.1009 \| \| AC091167.2 \| novel transcript \| -2.1009 \| \| AHRR \| aryl-hydrocarbon receptor repressor [Source:HGNC Symbol;Acc:HGNC:346] \| -2.03615 \| \| PCDHB2 \| protocadherin beta 2 [Source:HGNC Symbol;Acc:HGNC:8687] \| -2.0336 \| \| AC005256.1 \| novel transcript \| -2.01341 \| \| ULBP1 \| UL16 binding protein 1 [Source:HGNC Symbol;Acc:HGNC:14893] \| -2.01341 \| \| FAM131B \| family with sequence similarity 131 member B [Source:HGNC Symbol;Acc:HGNC:22202] \| -1.97072 \| \| HSH2D \| hematopoietic SH2 domain containing [Source:HGNC Symbol;Acc:HGNC:24920] \| -1.96764 \| \| GUSBP11 \| glucuronidase, beta pseudogene 11 [Source:HGNC Symbol;Acc:HGNC:42325] \| -1.96763 \| \| CSTA \| cystatin A [Source:HGNC Symbol;Acc:HGNC:2481] \| -1.96559 \| \| RDH12 \| retinol dehydrogenase 12 [Source:HGNC Symbol;Acc:HGNC:19977] \| -1.96088 \| \| MIEF2 \| mitochondrial elongation factor 2 [Source:HGNC Symbol;Acc:HGNC:17920] \| -1.95964 \| \| HNRNPUL2-BSCL2 \| HNRNPUL2-BSCL2 readthrough (NMD candidate) [Source:HGNC Symbol;Acc:HGNC:49189] \| -1.95533 \| \| SYS1-DBNDD2 \| SYS1-DBNDD2 readthrough (NMD candidate) [Source:HGNC Symbol;Acc:HGNC:33535] \| -1.9483 \| \| ZNF286A \| zinc finger protein 286A [Source:HGNC Symbol;Acc:HGNC:13501] \| -1.94153 \| \| IGFBP5 \| insulin like growth factor binding protein 5 [Source:HGNC Symbol;Acc:HGNC:5474] \| -1.92203 \| \| KCNH2 \| potassium voltage-gated channel subfamily H member 2 [Source:HGNC Symbol;Acc:HGNC:6251] \| -1.92032 \| \| C9orf106 \| chromosome 9 open reading frame 106 (putative) [Source:HGNC Symbol;Acc:HGNC:31370] \| -1.92026 \| \| TBX15 \| T-box 15 [Source:HGNC Symbol;Acc:HGNC:11594] \| -1.9139 \| \| RBM12 \| RNA binding motif protein 12 [Source:HGNC Symbol;Acc:HGNC:9898] \| -1.91244 \| \| GPD1L \| glycerol-3-phosphate dehydrogenase 1 like [Source:HGNC Symbol;Acc:HGNC:28956] \| -1.90714 \| \| PFN2 \| profilin 2 [Source:HGNC Symbol;Acc:HGNC:8882] \| -1.87878 \| \| UTS2 \| urotensin 2 [Source:HGNC Symbol;Acc:HGNC:12636] \| -1.87139 \| \| CRISP3 \| cysteine rich secretory protein 3 [Source:HGNC Symbol;Acc:HGNC:16904] \| -1.87139 \| \| CCDC114 \| coiled-coil domain containing 114 [Source:HGNC Symbol;Acc:HGNC:26560] \| -1.87139 \| \| QPRT \| quinolinate phosphoribosyltransferase [Source:HGNC Symbol;Acc:HGNC:9755] \| -1.87072 \| \| DNAL1 \| dynein axonemal light chain 1 [Source:HGNC Symbol;Acc:HGNC:23247] \| -1.86822 \| \| SHISAL1 \| shisa like 1 [Source:HGNC Symbol;Acc:HGNC:29335] \| -1.8449 \| \| GPC2 \| glypican 2 [Source:HGNC Symbol;Acc:HGNC:4450] \| -1.83941 \| \| MYH11 \| myosin heavy chain 11 [Source:HGNC Symbol;Acc:HGNC:7569] \| -1.83655 \| \| CREB5 \| cAMP responsive element binding protein 5 [Source:HGNC Symbol;Acc:HGNC:16844] \| -1.83121 \| \| GPX8 \| glutathione peroxidase 8 (putative) [Source:HGNC Symbol;Acc:HGNC:33100] \| -1.82518 \| \| DLX4 \| distal-less homeobox 4 [Source:HGNC Symbol;Acc:HGNC:2917] \| -1.82072 \| \| GSKIP \| GSK3B interacting protein [Source:HGNC Symbol;Acc:HGNC:20343] \| -1.81897 \| \| CD59 \| CD59 molecule (CD59 blood group) [Source:HGNC Symbol;Acc:HGNC:1689] \| -1.81533 \| \| CHAC1 \| ChaC glutathione specific gamma-glutamylcyclotransferase 1 [Source:HGNC Symbol;Acc:HGNC:28680] \| -1.80895 \| \| JAM3 \| junctional adhesion molecule 3 [Source:HGNC Symbol;Acc:HGNC:15532] \| -1.80599 \| \| RASA4B \| RAS p21 protein activator 4B [Source:HGNC Symbol;Acc:HGNC:35202] \| -1.78897 \| \| TMEM101 \| transmembrane protein 101 [Source:HGNC Symbol;Acc:HGNC:28653] \| -1.78896 \| \| SPIN1 \| spindlin 1 [Source:HGNC Symbol;Acc:HGNC:11243] \| -1.78635 \| \| LGI4 \| leucine rich repeat LGI family member 4 [Source:HGNC Symbol;Acc:HGNC:18712] \| -1.78212 \| \| ABAT \| 4-aminobutyrate aminotransferase [Source:HGNC Symbol;Acc:HGNC:23] \| -1.77893 \| \| FAM129A \| family with sequence similarity 129 member A [Source:HGNC Symbol;Acc:HGNC:16784] \| -1.77099 \| \| KLHL38 \| kelch like family member 38 [Source:HGNC Symbol;Acc:HGNC:34435] \| -1.76348 \| \| AP000866.1 \| uncharacterized LOC100507283 [Source:NCBI gene;Acc:100507283] \| -1.76348 \| \| MOAP1 \| modulator of apoptosis 1 [Source:HGNC Symbol;Acc:HGNC:16658] \| -1.76278 \| \| ADH1B \| alcohol dehydrogenase 1B (class I), beta polypeptide [Source:HGNC Symbol;Acc:HGNC:250] \| -1.76071 \| \| FAR1 \| fatty acyl-CoA reductase 1 [Source:HGNC Symbol;Acc:HGNC:26222] \| -1.75035 \| \| AC005839.1 \| TEC \| -1.75035 \| \| C1QL1 \| complement C1q like 1 [Source:HGNC Symbol;Acc:HGNC:24182] \| -1.73925 \| \| MIR4444-2 \| microRNA 4444-2 [Source:HGNC Symbol;Acc:HGNC:43481] \| -1.73365 \| \| NUPR1 \| nuclear protein 1, transcriptional regulator [Source:HGNC Symbol;Acc:HGNC:29990] \| -1.72708 \| \| DICER1-AS1 \| DICER1 antisense RNA 1 [Source:HGNC Symbol;Acc:HGNC:43017] \| -1.72394 \| \| CA9 \| carbonic anhydrase 9 [Source:HGNC Symbol;Acc:HGNC:1383] \| -1.70377 \| \| MMP13 \| matrix metallopeptidase 13 [Source:HGNC Symbol;Acc:HGNC:7159] \| -1.69931 \| \| AKIRIN1 \| akirin 1 [Source:HGNC Symbol;Acc:HGNC:25744] \| -1.69898 \| \| HSPE1-MOB4 \| HSPE1-MOB4 readthrough [Source:HGNC Symbol;Acc:HGNC:49184] \| -1.68803 \| \| ARL11 \| ADP ribosylation factor like GTPase 11 [Source:HGNC Symbol;Acc:HGNC:24046] \| -1.68585 \| \| FZD2 \| frizzled class receptor 2 [Source:HGNC Symbol;Acc:HGNC:4040] \| -1.6833 \| \| TTR \| transthyretin [Source:HGNC Symbol;Acc:HGNC:12405] \| -1.68328 \| \| RRN3P2 \| RRN3 homolog, RNA polymerase I transcription factor pseudogene 2 [Source:HGNC Symbol;Acc:HGNC:37619] \| -1.68179 \| \| LINC01239 \| long intergenic non-protein coding RNA 1239 [Source:HGNC Symbol;Acc:HGNC:49796] \| -1.6808 \| \| UNC5B \| unc-5 netrin receptor B [Source:HGNC Symbol;Acc:HGNC:12568] \| -1.68066 \| \| SRGAP3 \| SLIT-ROBO Rho GTPase activating protein 3 [Source:HGNC Symbol;Acc:HGNC:19744] \| -1.67636 \| \| GDF11 \| growth differentiation factor 11 [Source:HGNC Symbol;Acc:HGNC:4216] \| -1.66745 \| \| TMPRSS2 \| transmembrane serine protease 2 [Source:HGNC Symbol;Acc:HGNC:11876] \| -1.66324 \| \| GJA5 \| gap junction protein alpha 5 [Source:HGNC Symbol;Acc:HGNC:4279] \| -1.66224 \| \| AC074091.1 \| novel transcript \| -1.66111 \| \| SELENOI \| selenoprotein I [Source:HGNC Symbol;Acc:HGNC:29361] \| -1.65386 \| \| PHLDA3 \| pleckstrin homology like domain family A member 3 [Source:HGNC Symbol;Acc:HGNC:8934] \| -1.64306 \| \| SPRY1 \| sprouty RTK signaling antagonist 1 [Source:HGNC Symbol;Acc:HGNC:11269] \| -1.64219 \| \| PLA2G15 \| phospholipase A2 group XV [Source:HGNC Symbol;Acc:HGNC:17163] \| -1.6301 \| \| UNC13A \| unc-13 homolog A [Source:HGNC Symbol;Acc:HGNC:23150] \| -1.62749 \| \| PHGDH \| phosphoglycerate dehydrogenase [Source:HGNC Symbol;Acc:HGNC:8923] \| -1.62223 \| \| FXYD3 \| FXYD domain containing ion transport regulator 3 [Source:HGNC Symbol;Acc:HGNC:4027] \| -1.61332 \| \| MYCN \| MYCN proto-oncogene, bHLH transcription factor [Source:HGNC Symbol;Acc:HGNC:7559] \| -1.61167 \| \| CCDC106 \| coiled-coil domain containing 106 [Source:HGNC Symbol;Acc:HGNC:30181] \| -1.5984 \| \| CCL15 \| C-C motif chemokine ligand 15 [Source:HGNC Symbol;Acc:HGNC:10613] \| -1.59838 \| \| AC009133.1 \| novel transcript, antisense to C16orf53 and MVP \| -1.59836 \| \| IKZF2 \| IKAROS family zinc finger 2 [Source:HGNC Symbol;Acc:HGNC:13177] \| -1.59835 \| \| AC084757.1 \| oxidation resistance 1 (OXR1) pseudogene \| -1.59833 \| \| GSDMB \| gasdermin B [Source:HGNC Symbol;Acc:HGNC:23690] \| -1.58893 \| \| COL5A1 \| collagen type V alpha 1 chain [Source:HGNC Symbol;Acc:HGNC:2209] \| -1.58566 \| \| CBS \| cystathionine-beta-synthase [Source:HGNC Symbol;Acc:HGNC:1550] \| -1.58396 \| \| ZNF583 \| zinc finger protein 583 [Source:HGNC Symbol;Acc:HGNC:26427] \| -1.58329 \| \| SCNN1A \| sodium channel epithelial 1 alpha subunit [Source:HGNC Symbol;Acc:HGNC:10599] \| -1.57806 \| \| AC003102.1 \| novel transcript, antisense to UBTF \| -1.57142 \| \| GACAT2 \| gastric cancer associated transcript 2 [Source:HGNC Symbol;Acc:HGNC:50516] \| -1.57142 \| \| ST6GALNAC1 \| ST6 N-acetylgalactosaminide alpha-2,6-sialyltransferase 1 [Source:HGNC Symbol;Acc:HGNC:23614] \| -1.56979 \| \| NDUFA4L2 \| NDUFA4, mitochondrial complex associated like 2 [Source:HGNC Symbol;Acc:HGNC:29836] \| -1.56863 \| \| KIAA1211L \| KIAA1211 like [Source:HGNC Symbol;Acc:HGNC:33454] \| -1.56804 \| \| TET1 \| tet methylcytosine dioxygenase 1 [Source:HGNC Symbol;Acc:HGNC:29484] \| -1.56804 \| \| TMEM133 \| - \| -1.56367 \| \| KIAA0232 \| KIAA0232 [Source:HGNC Symbol;Acc:HGNC:28992] \| -1.56312 \| \| SPARCL1 \| SPARC like 1 [Source:HGNC Symbol;Acc:HGNC:11220] \| -1.56149 \| \| CRIP1 \| cysteine rich protein 1 [Source:HGNC Symbol;Acc:HGNC:2360] \| -1.56093 \| \| ZDHHC8P1 \| zinc finger DHHC-type containing 8 pseudogene 1 [Source:HGNC Symbol;Acc:HGNC:26461] \| -1.55658 \| \| PSAT1 \| phosphoserine aminotransferase 1 [Source:HGNC Symbol;Acc:HGNC:19129] \| -1.55616 \| \| ATP2A3 \| ATPase sarcoplasmic/endoplasmic reticulum Ca2+ transporting 3 [Source:HGNC Symbol;Acc:HGNC:813] \| -1.55532 \| \| AL591379.2 \| pseudogene similar to part of tripartite motif-containing 43 (TRIM43) \| -1.55259 \| \| FHL1 \| four and a half LIM domains 1 [Source:HGNC Symbol;Acc:HGNC:3702] \| -1.54108 \| \| STEAP2 \| STEAP2 metalloreductase [Source:HGNC Symbol;Acc:HGNC:17885] \| -1.5384 \| \| DUOX2 \| dual oxidase 2 [Source:HGNC Symbol;Acc:HGNC:13273] \| -1.537 \| \| CBSL \| cystathionine-beta-synthase like [Source:HGNC Symbol;Acc:HGNC:51829] \| -1.53426 \| \| CCDC92 \| coiled-coil domain containing 92 [Source:HGNC Symbol;Acc:HGNC:29563] \| -1.53038 \| \| PCK2 \| phosphoenolpyruvate carboxykinase 2, mitochondrial [Source:HGNC Symbol;Acc:HGNC:8725] \| -1.52334 \| \| MAP1LC3B \| microtubule associated protein 1 light chain 3 beta [Source:HGNC Symbol;Acc:HGNC:13352] \| -1.52309 \| \| IL17RD \| interleukin 17 receptor D [Source:HGNC Symbol;Acc:HGNC:17616] \| -1.51087 \| \| IGFBP1 \| insulin like growth factor binding protein 1 [Source:HGNC Symbol;Acc:HGNC:5469] \| -1.50575 \| \| RNF185 \| ring finger protein 185 [Source:HGNC Symbol;Acc:HGNC:26783] \| -1.50422 \| \| FSTL5 \| follistatin like 5 [Source:HGNC Symbol;Acc:HGNC:21386] \| -1.49882 \| \| TJP3 \| tight junction protein 3 [Source:HGNC Symbol;Acc:HGNC:11829] \| -1.49341 \| \| ABCG1 \| ATP binding cassette subfamily G member 1 [Source:HGNC Symbol;Acc:HGNC:73] \| -1.49147 \| \| ACVR1B \| activin A receptor type 1B [Source:HGNC Symbol;Acc:HGNC:172] \| -1.49011 \| \| SLC6A9 \| solute carrier family 6 member 9 [Source:HGNC Symbol;Acc:HGNC:11056] \| -1.48906 \| \| NELL1 \| neural EGFL like 1 [Source:HGNC Symbol;Acc:HGNC:7750] \| -1.48833 \| \| DACT2 \| dishevelled binding antagonist of beta catenin 2 [Source:HGNC Symbol;Acc:HGNC:21231] \| -1.48617 \| \| COLCA1 \| colorectal cancer associated 1 [Source:HGNC Symbol;Acc:HGNC:33789] \| -1.48128 \| \| SENP3 \| SUMO specific peptidase 3 [Source:HGNC Symbol;Acc:HGNC:17862] \| -1.47571 \| \| LINC01393 \| long intergenic non-protein coding RNA 1393 [Source:HGNC Symbol;Acc:HGNC:50669] \| -1.47553 \| \| GBGT1 \| globoside alpha-1,3-N-acetylgalactosaminyltransferase 1 (FORS blood group) [Source:HGNC Symbol;Acc:HGNC:20460] \| -1.47288 \| \| FCGBP \| Fc fragment of IgG binding protein [Source:HGNC Symbol;Acc:HGNC:13572] \| -1.46512 \| \| AC090527.2 \| novel protein \| -1.4641 \| \| NEURL1B \| neuralized E3 ubiquitin protein ligase 1B [Source:HGNC Symbol;Acc:HGNC:35422] \| -1.46294 \| \| DUSP3 \| dual specificity phosphatase 3 [Source:HGNC Symbol;Acc:HGNC:3069] \| -1.46018 \| \| MOGS \| mannosyl-oligosaccharide glucosidase [Source:HGNC Symbol;Acc:HGNC:24862] \| -1.45562 \| \| TRIM26 \| tripartite motif containing 26 [Source:HGNC Symbol;Acc:HGNC:12962] \| -1.45426 \| \| ZNF385B \| zinc finger protein 385B [Source:HGNC Symbol;Acc:HGNC:26332] \| -1.45085 \| \| PDXDC2P \| pyridoxal dependent decarboxylase domain containing 2, pseudogene [Source:HGNC Symbol;Acc:HGNC:27559] \| -1.44838 \| \| DYRK1B \| dual specificity tyrosine phosphorylation regulated kinase 1B [Source:HGNC Symbol;Acc:HGNC:3092] \| -1.44498 \| \| SLX1A-SULT1A3 \| SLX1A-SULT1A3 readthrough (NMD candidate) [Source:HGNC Symbol;Acc:HGNC:44437] \| -1.44226 \| \| HKR1 \| HKR1, GLI-Kruppel zinc finger family member [Source:HGNC Symbol;Acc:HGNC:4928] \| -1.44152 \| \| ZNF155 \| zinc finger protein 155 [Source:HGNC Symbol;Acc:HGNC:12940] \| -1.43611 \| \| ADH1C \| alcohol dehydrogenase 1C (class I), gamma polypeptide [Source:HGNC Symbol;Acc:HGNC:251] \| -1.43469 \| \| HES6 \| hes family bHLH transcription factor 6 [Source:HGNC Symbol;Acc:HGNC:18254] \| -1.43375 \| \| BCAT1 \| branched chain amino acid transaminase 1 [Source:HGNC Symbol;Acc:HGNC:976] \| -1.43251 \| \| CFB \| complement factor B [Source:HGNC Symbol;Acc:HGNC:1037] \| -1.42331 \| \| AL121845.3 \| novel protein, ZGPAT-LIME1 readthrough \| -1.42331 \| \| BTF3L4 \| basic transcription factor 3 like 4 [Source:HGNC Symbol;Acc:HGNC:30547] \| -1.42311 \| \| SAT1 \| spermidine/spermine N1-acetyltransferase 1 [Source:HGNC Symbol;Acc:HGNC:10540] \| -1.42291 \| \| ASNS \| asparagine synthetase (glutamine-hydrolyzing) [Source:HGNC Symbol;Acc:HGNC:753] \| -1.41863 \| \| BHLHB9 \| basic helix-loop-helix family member b9 [Source:HGNC Symbol;Acc:HGNC:29353] \| -1.41817 \| \| SATB1 \| SATB homeobox 1 [Source:HGNC Symbol;Acc:HGNC:10541] \| -1.41777 \| \| SPTSSB \| serine palmitoyltransferase small subunit B [Source:HGNC Symbol;Acc:HGNC:24045] \| -1.41617 \| \| GHDC \| GH3 domain containing [Source:HGNC Symbol;Acc:HGNC:24438] \| -1.412 \| \| MANSC1 \| MANSC domain containing 1 [Source:HGNC Symbol;Acc:HGNC:25505] \| -1.41 \| \| MPZL2 \| myelin protein zero like 2 [Source:HGNC Symbol;Acc:HGNC:3496] \| -1.4077 \| \| ZNF513 \| zinc finger protein 513 [Source:HGNC Symbol;Acc:HGNC:26498] \| -1.40451 \| \| AC010422.3 \| novel transcript \| -1.39809 \| \| HVCN1 \| hydrogen voltage gated channel 1 [Source:HGNC Symbol;Acc:HGNC:28240] \| -1.39755 \| \| TMEM128 \| transmembrane protein 128 [Source:HGNC Symbol;Acc:HGNC:28201] \| -1.39506 \| \| OLFML2B \| olfactomedin like 2B [Source:HGNC Symbol;Acc:HGNC:24558] \| -1.39191 \| \| ASS1 \| argininosuccinate synthase 1 [Source:HGNC Symbol;Acc:HGNC:758] \| -1.39087 \| \| ZNF286B \| zinc finger protein 286B [Source:HGNC Symbol;Acc:HGNC:33241] \| -1.38739 \| \| PTGER2 \| prostaglandin E receptor 2 [Source:HGNC Symbol;Acc:HGNC:9594] \| -1.3869 \| \| SYNGR4 \| synaptogyrin 4 [Source:HGNC Symbol;Acc:HGNC:11502] \| -1.38581 \| \| SULT1C4 \| sulfotransferase family 1C member 4 [Source:HGNC Symbol;Acc:HGNC:11457] \| -1.38426 \| \| POLR2M \| RNA polymerase II subunit M [Source:HGNC Symbol;Acc:HGNC:14862] \| -1.38379 \| \| MUC5B \| mucin 5B, oligomeric mucus/gel-forming [Source:HGNC Symbol;Acc:HGNC:7516] \| -1.3814 \| \| CHST11 \| carbohydrate sulfotransferase 11 [Source:HGNC Symbol;Acc:HGNC:17422] \| -1.38034 \| \| PDK2 \| pyruvate dehydrogenase kinase 2 [Source:HGNC Symbol;Acc:HGNC:8810] \| -1.37956 \| \| RIMS3 \| regulating synaptic membrane exocytosis 3 [Source:HGNC Symbol;Acc:HGNC:21292] \| -1.37769 \| \| MACROD1 \| MACRO domain containing 1 [Source:HGNC Symbol;Acc:HGNC:29598] \| -1.37746 \| \| PCMTD1 \| protein-L-isoaspartate (D-aspartate) O-methyltransferase domain containing 1 [Source:HGNC Symbol;Acc:HGNC:30483] \| -1.37466 \| \| CYB5RL \| cytochrome b5 reductase like [Source:HGNC Symbol;Acc:HGNC:32220] \| -1.3687 \| \| SNX10 \| sorting nexin 10 [Source:HGNC Symbol;Acc:HGNC:14974] \| -1.36826 \| \| RCBTB1 \| RCC1 and BTB domain containing protein 1 [Source:HGNC Symbol;Acc:HGNC:18243] \| -1.36317 \| \| PIGR \| polymeric immunoglobulin receptor [Source:HGNC Symbol;Acc:HGNC:8968] \| -1.36138 \| \| MYO7A \| myosin VIIA [Source:HGNC Symbol;Acc:HGNC:7606] \| -1.36135 \| \| ZBED6 \| zinc finger BED-type containing 6 [Source:HGNC Symbol;Acc:HGNC:33273] \| -1.3577 \| \| FMOD \| fibromodulin [Source:HGNC Symbol;Acc:HGNC:3774] \| -1.35047 \| \| CXXC5 \| CXXC finger protein 5 [Source:HGNC Symbol;Acc:HGNC:26943] \| -1.34949 \| \| ZNF260 \| zinc finger protein 260 [Source:HGNC Symbol;Acc:HGNC:13499] \| -1.34839 \| \| ZNF107 \| zinc finger protein 107 [Source:HGNC Symbol;Acc:HGNC:12887] \| -1.34753 \| \| BLOC1S6 \| biogenesis of lysosomal organelles complex 1 subunit 6 [Source:HGNC Symbol;Acc:HGNC:8549] \| -1.34706 \| \| KCNT2 \| potassium sodium-activated channel subfamily T member 2 [Source:HGNC Symbol;Acc:HGNC:18866] \| -1.34359 \| \| AC073136.1 \| exportin, tRNA (nuclear export receptor for tRNAs) (XPOT) pseudogene \| -1.33612 \| \| MXD1 \| MAX dimerization protein 1 [Source:HGNC Symbol;Acc:HGNC:6761] \| -1.33173 \| \| TGFBR1 \| transforming growth factor beta receptor 1 [Source:HGNC Symbol;Acc:HGNC:11772] \| -1.32737 \| \| TMED1 \| transmembrane p24 trafficking protein 1 [Source:HGNC Symbol;Acc:HGNC:17291] \| -1.32658 \| \| ZSCAN31 \| zinc finger and SCAN domain containing 31 [Source:HGNC Symbol;Acc:HGNC:14097] \| -1.32548 \| \| ORAI3 \| ORAI calcium release-activated calcium modulator 3 [Source:HGNC Symbol;Acc:HGNC:28185] \| -1.32086 \| \| STMN1 \| stathmin 1 [Source:HGNC Symbol;Acc:HGNC:6510] \| -1.32067 \| \| SLCO2A1 \| solute carrier organic anion transporter family member 2A1 [Source:HGNC Symbol;Acc:HGNC:10955] \| -1.31602 \| \| WDTC1 \| WD and tetratricopeptide repeats 1 [Source:HGNC Symbol;Acc:HGNC:29175] \| -1.31276 \| \| ZNF652 \| zinc finger protein 652 [Source:HGNC Symbol;Acc:HGNC:29147] \| -1.31273 \| \| TRAPPC2 \| trafficking protein particle complex 2 [Source:HGNC Symbol;Acc:HGNC:23068] \| -1.31227 \| \| LASP1 \| LIM and SH3 protein 1 [Source:HGNC Symbol;Acc:HGNC:6513] \| -1.31214 \| \| NBPF3 \| NBPF member 3 [Source:HGNC Symbol;Acc:HGNC:25076] \| -1.31177 \| \| PRRG4 \| proline rich and Gla domain 4 [Source:HGNC Symbol;Acc:HGNC:30799] \| -1.30886 \| \| ANKRD11 \| ankyrin repeat domain 11 [Source:HGNC Symbol;Acc:HGNC:21316] \| -1.30876 \| \| ZFP62 \| ZFP62 zinc finger protein [Source:HGNC Symbol;Acc:HGNC:23241] \| -1.30766 \| \| LGALS9 \| galectin 9 [Source:HGNC Symbol;Acc:HGNC:6570] \| -1.30741 \| \| RAB37 \| RAB37, member RAS oncogene family [Source:HGNC Symbol;Acc:HGNC:30268] \| -1.3062 \| \| ZBTB46 \| zinc finger and BTB domain containing 46 [Source:HGNC Symbol;Acc:HGNC:16094] \| -1.30605 \| \| GATM \| glycine amidinotransferase [Source:HGNC Symbol;Acc:HGNC:4175] \| -1.30156 \| \| C4BPA \| complement component 4 binding protein alpha [Source:HGNC Symbol;Acc:HGNC:1325] \| -1.29444 \| \| HSF2BP \| heat shock transcription factor 2 binding protein [Source:HGNC Symbol;Acc:HGNC:5226] \| -1.29025 \| \| BIRC7 \| baculoviral IAP repeat containing 7 [Source:HGNC Symbol;Acc:HGNC:13702] \| -1.28645 \| \| IFITM2 \| interferon induced transmembrane protein 2 [Source:HGNC Symbol;Acc:HGNC:5413] \| -1.28524 \| \| FP236383.1 \| novel transcript \| -1.27646 \| \| VILL \| villin like [Source:HGNC Symbol;Acc:HGNC:30906] \| -1.27643 \| \| HMGCS2 \| 3-hydroxy-3-methylglutaryl-CoA synthase 2 [Source:HGNC Symbol;Acc:HGNC:5008] \| -1.27367 \| \| TMEM176A \| transmembrane protein 176A [Source:HGNC Symbol;Acc:HGNC:24930] \| -1.27121 \| \| C21orf58 \| chromosome 21 open reading frame 58 [Source:HGNC Symbol;Acc:HGNC:1300] \| -1.26925 \| \| MGP \| matrix Gla protein [Source:HGNC Symbol;Acc:HGNC:7060] \| -1.26868 \| \| PROS1 \| protein S [Source:HGNC Symbol;Acc:HGNC:9456] \| -1.26774 \| \| ITGB4 \| integrin subunit beta 4 [Source:HGNC Symbol;Acc:HGNC:6158] \| -1.26458 \| \| BHMT \| betaine--homocysteine S-methyltransferase [Source:HGNC Symbol;Acc:HGNC:1047] \| -1.26136 \| \| UBC \| ubiquitin C [Source:HGNC Symbol;Acc:HGNC:12468] \| -1.25924 \| \| CDKN2C \| cyclin dependent kinase inhibitor 2C [Source:HGNC Symbol;Acc:HGNC:1789] \| -1.25698 \| \| REST \| RE1 silencing transcription factor [Source:HGNC Symbol;Acc:HGNC:9966] \| -1.25443 \| \| IKZF4 \| IKAROS family zinc finger 4 [Source:HGNC Symbol;Acc:HGNC:13179] \| -1.25262 \| \| RNPEPL1 \| arginyl aminopeptidase like 1 [Source:HGNC Symbol;Acc:HGNC:10079] \| -1.25148 \| \| MXD3 \| MAX dimerization protein 3 [Source:HGNC Symbol;Acc:HGNC:14008] \| -1.24788 \| \| S100A10 \| S100 calcium binding protein A10 [Source:HGNC Symbol;Acc:HGNC:10487] \| -1.24229 \| \| DNPH1 \| 2'-deoxynucleoside 5'-phosphate N-hydrolase 1 [Source:HGNC Symbol;Acc:HGNC:21218] \| -1.2385 \| \| BAIAP3 \| BAI1 associated protein 3 [Source:HGNC Symbol;Acc:HGNC:948] \| -1.23581 \| \| PTGIS \| prostaglandin I2 synthase [Source:HGNC Symbol;Acc:HGNC:9603] \| -1.23226 \| \| AC107871.1 \| novel protein \| -1.23046 \| \| AP002373.1 \| novel transcript, RBM7-REXO2 readthrough \| -1.22844 \| \| CD93 \| CD93 molecule [Source:HGNC Symbol;Acc:HGNC:15855] \| -1.22843 \| \| PITPNB \| phosphatidylinositol transfer protein beta [Source:HGNC Symbol;Acc:HGNC:9002] \| -1.22742 \| \| CCNG2 \| cyclin G2 [Source:HGNC Symbol;Acc:HGNC:1593] \| -1.22154 \| \| GPD1 \| glycerol-3-phosphate dehydrogenase 1 [Source:HGNC Symbol;Acc:HGNC:4455] \| -1.21986 \| \| SLC6A16 \| solute carrier family 6 member 16 [Source:HGNC Symbol;Acc:HGNC:13622] \| -1.21986 \| \| CP \| ceruloplasmin [Source:HGNC Symbol;Acc:HGNC:2295] \| -1.21242 \| \| NHSL2 \| NHS like 2 [Source:HGNC Symbol;Acc:HGNC:33737] \| -1.21201 \| \| ITGB8 \| integrin subunit beta 8 [Source:HGNC Symbol;Acc:HGNC:6163] \| -1.21047 \| \| HABP2 \| hyaluronan binding protein 2 [Source:HGNC Symbol;Acc:HGNC:4798] \| -1.20897 \| \| KLHL24 \| kelch like family member 24 [Source:HGNC Symbol;Acc:HGNC:25947] \| -1.20449 \| \| ERBB3 \| erb-b2 receptor tyrosine kinase 3 [Source:HGNC Symbol;Acc:HGNC:3431] \| -1.20427 \| \| HMGB2 \| high mobility group box 2 [Source:HGNC Symbol;Acc:HGNC:5000] \| -1.20343 \| \| RORA \| RAR related orphan receptor A [Source:HGNC Symbol;Acc:HGNC:10258] \| -1.20326 \| \| COCH \| cochlin [Source:HGNC Symbol;Acc:HGNC:2180] \| -1.19629 \| \| MTAP \| methylthioadenosine phosphorylase [Source:HGNC Symbol;Acc:HGNC:7413] \| -1.19333 \| \| ROM1 \| retinal outer segment membrane protein 1 [Source:HGNC Symbol;Acc:HGNC:10254] \| -1.19175 \| \| IGLC2 \| immunoglobulin lambda constant 2 [Source:HGNC Symbol;Acc:HGNC:5856] \| -1.18975 \| \| CIAO2B \| cytosolic iron-sulfur assembly component 2B [Source:HGNC Symbol;Acc:HGNC:24261] \| -1.18115 \| \| ARL8B \| ADP ribosylation factor like GTPase 8B [Source:HGNC Symbol;Acc:HGNC:25564] \| -1.17773 \| \| ADAP1 \| ArfGAP with dual PH domains 1 [Source:HGNC Symbol;Acc:HGNC:16486] \| -1.17692 \| \| COL21A1 \| collagen type XXI alpha 1 chain [Source:HGNC Symbol;Acc:HGNC:17025] \| -1.17487 \| \| FBP1 \| fructose-bisphosphatase 1 [Source:HGNC Symbol;Acc:HGNC:3606] \| -1.17389 \| \| CYBB \| cytochrome b-245 beta chain [Source:HGNC Symbol;Acc:HGNC:2578] \| -1.17389 \| \| VCAN \| versican [Source:HGNC Symbol;Acc:HGNC:2464] \| -1.17314 \| \| SPECC1L-ADORA2A \| SPECC1L-ADORA2A readthrough (NMD candidate) [Source:HGNC Symbol;Acc:HGNC:49185] \| -1.17226 \| \| SESN3 \| sestrin 3 [Source:HGNC Symbol;Acc:HGNC:23060] \| -1.17096 \| \| CGNL1 \| cingulin like 1 [Source:HGNC Symbol;Acc:HGNC:25931] \| -1.17025 \| \| ACSF2 \| acyl-CoA synthetase family member 2 [Source:HGNC Symbol;Acc:HGNC:26101] \| -1.17022 \| \| ITGA10 \| integrin subunit alpha 10 [Source:HGNC Symbol;Acc:HGNC:6135] \| -1.16954 \| \| LRAT \| lecithin retinol acyltransferase [Source:HGNC Symbol;Acc:HGNC:6685] \| -1.16921 \| \| TMEM219 \| transmembrane protein 219 [Source:HGNC Symbol;Acc:HGNC:25201] \| -1.16655 \| \| CYP24A1 \| cytochrome P450 family 24 subfamily A member 1 [Source:HGNC Symbol;Acc:HGNC:2602] \| -1.1663 \| \| CEACAM5 \| carcinoembryonic antigen related cell adhesion molecule 5 [Source:HGNC Symbol;Acc:HGNC:1817] \| -1.1657 \| \| PGM5P2 \| phosphoglucomutase 5 pseudogene 2 [Source:HGNC Symbol;Acc:HGNC:18965] \| -1.16542 \| \| FAM189B \| family with sequence similarity 189 member B [Source:HGNC Symbol;Acc:HGNC:1233] \| -1.16436 \| \| TCEAL4 \| transcription elongation factor A like 4 [Source:HGNC Symbol;Acc:HGNC:26121] \| -1.1638 \| \| POC1B-GALNT4 \| POC1B-GALNT4 readthrough [Source:HGNC Symbol;Acc:HGNC:42957] \| -1.16316 \| \| RTN3 \| reticulon 3 [Source:HGNC Symbol;Acc:HGNC:10469] \| -1.15899 \| \| C7orf25 \| chromosome 7 open reading frame 25 [Source:HGNC Symbol;Acc:HGNC:21703] \| -1.15837 \| \| HHLA2 \| HERV-H LTR-associating 2 [Source:HGNC Symbol;Acc:HGNC:4905] \| -1.15485 \| \| CREBRF \| CREB3 regulatory factor [Source:HGNC Symbol;Acc:HGNC:24050] \| -1.15249 \| \| ZNF619 \| zinc finger protein 619 [Source:HGNC Symbol;Acc:HGNC:26910] \| -1.15092 \| \| AP003108.2 \| novel transcript \| -1.14808 \| \| CTH \| cystathionine gamma-lyase [Source:HGNC Symbol;Acc:HGNC:2501] \| -1.14747 \| \| TMEM150A \| transmembrane protein 150A [Source:HGNC Symbol;Acc:HGNC:24677] \| -1.14467 \| \| ZFP69B \| ZFP69 zinc finger protein B [Source:HGNC Symbol;Acc:HGNC:28053] \| -1.14467 \| \| MPP7 \| membrane palmitoylated protein 7 [Source:HGNC Symbol;Acc:HGNC:26542] \| -1.14382 \| \| AC021054.1 \| uncharacterized LOC100049716 [Source:NCBI gene;Acc:100049716] \| -1.14363 \| \| DDIT4 \| DNA damage inducible transcript 4 [Source:HGNC Symbol;Acc:HGNC:24944] \| -1.14352 \| \| DOC2B \| double C2 domain beta [Source:HGNC Symbol;Acc:HGNC:2986] \| -1.14331 \| \| MMP9 \| matrix metallopeptidase 9 [Source:HGNC Symbol;Acc:HGNC:7176] \| -1.14271 \| \| AP000919.1 \| cysteine and histidine-rich domain (CHORD) containing 1 (CHORDC1) pseudogene \| -1.14153 \| \| LINC01234 \| long intergenic non-protein coding RNA 1234 [Source:HGNC Symbol;Acc:HGNC:49757] \| -1.14052 \| \| STRA6LP \| STRA6 like, pseudogene [Source:HGNC Symbol;Acc:HGNC:53830] \| -1.13629 \| \| SCRN2 \| secernin 2 [Source:HGNC Symbol;Acc:HGNC:30381] \| -1.13576 \| \| GPRIN3 \| GPRIN family member 3 [Source:HGNC Symbol;Acc:HGNC:27733] \| -1.13444 \| \| ITM2A \| integral membrane protein 2A [Source:HGNC Symbol;Acc:HGNC:6173] \| -1.13004 \| \| MFSD3 \| major facilitator superfamily domain containing 3 [Source:HGNC Symbol;Acc:HGNC:25157] \| -1.12688 \| \| AC068533.4 \| novel protein \| -1.12633 \| \| MED21 \| mediator complex subunit 21 [Source:HGNC Symbol;Acc:HGNC:11473] \| -1.12517 \| \| TP53INP1 \| tumor protein p53 inducible nuclear protein 1 [Source:HGNC Symbol;Acc:HGNC:18022] \| -1.1231 \| \| ADAMTS10 \| ADAM metallopeptidase with thrombospondin type 1 motif 10 [Source:HGNC Symbol;Acc:HGNC:13201] \| -1.12305 \| \| FOXO4 \| forkhead box O4 [Source:HGNC Symbol;Acc:HGNC:7139] \| -1.12226 \| \| AC104966.1 \| ceruloplasmin (ferroxidase) (CP) pseudogene \| -1.11758 \| \| ANKRD50 \| ankyrin repeat domain 50 [Source:HGNC Symbol;Acc:HGNC:29223] \| -1.1164 \| \| NRBP2 \| nuclear receptor binding protein 2 [Source:HGNC Symbol;Acc:HGNC:19339] \| -1.11385 \| \| LINC01426 \| long intergenic non-protein coding RNA 1426 [Source:HGNC Symbol;Acc:HGNC:50734] \| -1.11297 \| \| NUDT3 \| nudix hydrolase 3 [Source:HGNC Symbol;Acc:HGNC:8050] \| -1.11204 \| \| UAP1L1 \| UDP-N-acetylglucosamine pyrophosphorylase 1 like 1 [Source:HGNC Symbol;Acc:HGNC:28082] \| -1.11179 \| \| NEK9 \| NIMA related kinase 9 [Source:HGNC Symbol;Acc:HGNC:18591] \| -1.11036 \| \| DEFB1 \| defensin beta 1 [Source:HGNC Symbol;Acc:HGNC:2766] \| -1.10964 \| \| HMG20B \| high mobility group 20B [Source:HGNC Symbol;Acc:HGNC:5002] \| -1.10886 \| \| PTGER3 \| prostaglandin E receptor 3 [Source:HGNC Symbol;Acc:HGNC:9595] \| -1.10859 \| \| MAPT \| microtubule associated protein tau [Source:HGNC Symbol;Acc:HGNC:6893] \| -1.10794 \| \| UBA7 \| ubiquitin like modifier activating enzyme 7 [Source:HGNC Symbol;Acc:HGNC:12471] \| -1.10224 \| \| AC068631.3 \| PET100 homolog (PET100) pseudogene \| -1.10089 \| \| NKTR \| natural killer cell triggering receptor [Source:HGNC Symbol;Acc:HGNC:7833] \| -1.10019 \| \| TMEM38A \| transmembrane protein 38A [Source:HGNC Symbol;Acc:HGNC:28462] \| -1.09915 \| \| CHD5 \| chromodomain helicase DNA binding protein 5 [Source:HGNC Symbol;Acc:HGNC:16816] \| -1.09749 \| \| NECAP2 \| NECAP endocytosis associated 2 [Source:HGNC Symbol;Acc:HGNC:25528] \| -1.09542 \| \| YPEL3 \| yippee like 3 [Source:HGNC Symbol;Acc:HGNC:18327] \| -1.09434 \| \| C5AR2 \| complement component 5a receptor 2 [Source:HGNC Symbol;Acc:HGNC:4527] \| -1.09144 \| \| RAB3C \| RAB3C, member RAS oncogene family [Source:HGNC Symbol;Acc:HGNC:30269] \| -1.08937 \| \| MXI1 \| MAX interactor 1, dimerization protein [Source:HGNC Symbol;Acc:HGNC:7534] \| -1.08928 \| \| AKT1S1 \| AKT1 substrate 1 [Source:HGNC Symbol;Acc:HGNC:28426] \| -1.08812 \| \| PGM5 \| phosphoglucomutase 5 [Source:HGNC Symbol;Acc:HGNC:8908] \| -1.0864 \| \| KCNMB4 \| potassium calcium-activated channel subfamily M regulatory beta subunit 4 [Source:HGNC Symbol;Acc:HGNC:6289] \| -1.08468 \| \| FRY \| FRY microtubule binding protein [Source:HGNC Symbol;Acc:HGNC:20367] \| -1.08416 \| \| YIF1B \| Yip1 interacting factor homolog B, membrane trafficking protein [Source:HGNC Symbol;Acc:HGNC:30511] \| -1.08416 \| \| LBX2 \| ladybird homeobox 2 [Source:HGNC Symbol;Acc:HGNC:15525] \| -1.08381 \| \| TESC \| tescalcin [Source:HGNC Symbol;Acc:HGNC:26065] \| -1.08054 \| \| MEIS3 \| Meis homeobox 3 [Source:HGNC Symbol;Acc:HGNC:29537] \| -1.0804 \| \| SYTL5 \| synaptotagmin like 5 [Source:HGNC Symbol;Acc:HGNC:15589] \| -1.07946 \| \| RTN2 \| reticulon 2 [Source:HGNC Symbol;Acc:HGNC:10468] \| -1.07885 \| \| TMEM139 \| transmembrane protein 139 [Source:HGNC Symbol;Acc:HGNC:22058] \| -1.07519 \| \| ZNF511-PRAP1 \| ZNF511-PRAP1 readthrough [Source:HGNC Symbol;Acc:HGNC:38088] \| -1.07056 \| \| ALDH6A1 \| aldehyde dehydrogenase 6 family member A1 [Source:HGNC Symbol;Acc:HGNC:7179] \| -1.06686 \| \| HDGF \| heparin binding growth factor [Source:HGNC Symbol;Acc:HGNC:4856] \| -1.06392 \| \| CEMIP \| cell migration inducing hyaluronidase 1 [Source:HGNC Symbol;Acc:HGNC:29213] \| -1.0631 \| \| CCPG1 \| cell cycle progression 1 [Source:HGNC Symbol;Acc:HGNC:24227] \| -1.06251 \| \| GAMT \| guanidinoacetate N-methyltransferase [Source:HGNC Symbol;Acc:HGNC:4136] \| -1.06126 \| \| ZHX1 \| zinc fingers and homeoboxes 1 [Source:HGNC Symbol;Acc:HGNC:12871] \| -1.05756 \| \| PRDM8 \| PR/SET domain 8 [Source:HGNC Symbol;Acc:HGNC:13993] \| -1.05429 \| \| TRIM2 \| tripartite motif containing 2 [Source:HGNC Symbol;Acc:HGNC:15974] \| -1.05412 \| \| GDF15 \| growth differentiation factor 15 [Source:HGNC Symbol;Acc:HGNC:30142] \| -1.05372 \| \| CNEP1R1 \| CTD nuclear envelope phosphatase 1 regulatory subunit 1 [Source:HGNC Symbol;Acc:HGNC:26759] \| -1.05355 \| \| DUSP18 \| dual specificity phosphatase 18 [Source:HGNC Symbol;Acc:HGNC:18484] \| -1.0535 \| \| PIF1 \| PIF1 5'-to-3' DNA helicase [Source:HGNC Symbol;Acc:HGNC:26220] \| -1.05294 \| \| BTBD2 \| BTB domain containing 2 [Source:HGNC Symbol;Acc:HGNC:15504] \| -1.04575 \| \| ANXA9 \| annexin A9 [Source:HGNC Symbol;Acc:HGNC:547] \| -1.04359 \| \| GCNT2 \| glucosaminyl (N-acetyl) transferase 2 (I blood group) [Source:HGNC Symbol;Acc:HGNC:4204] \| -1.04062 \| \| CTSO \| cathepsin O [Source:HGNC Symbol;Acc:HGNC:2542] \| -1.04052 \| \| MUC15 \| mucin 15, cell surface associated [Source:HGNC Symbol;Acc:HGNC:14956] \| -1.03842 \| \| ZNF91 \| zinc finger protein 91 [Source:HGNC Symbol;Acc:HGNC:13166] \| -1.03727 \| \| FOXN3 \| forkhead box N3 [Source:HGNC Symbol;Acc:HGNC:1928] \| -1.03612 \| \| PCED1A \| PC-esterase domain containing 1A [Source:HGNC Symbol;Acc:HGNC:16212] \| -1.03579 \| \| APH1B \| aph-1 homolog B, gamma-secretase subunit [Source:HGNC Symbol;Acc:HGNC:24080] \| -1.03578 \| \| TMEM132A \| transmembrane protein 132A [Source:HGNC Symbol;Acc:HGNC:31092] \| -1.03462 \| \| TNFAIP2 \| TNF alpha induced protein 2 [Source:HGNC Symbol;Acc:HGNC:11895] \| -1.03451 \| \| CHST15 \| carbohydrate sulfotransferase 15 [Source:HGNC Symbol;Acc:HGNC:18137] \| -1.03347 \| \| PRXL2B \| peroxiredoxin like 2B [Source:HGNC Symbol;Acc:HGNC:28390] \| -1.02945 \| \| RHOQP2 \| ras homolog family member Q pseudogene 2 [Source:HGNC Symbol;Acc:HGNC:37836] \| -1.02919 \| \| FUCA1 \| alpha-L-fucosidase 1 [Source:HGNC Symbol;Acc:HGNC:4006] \| -1.0252 \| \| SLC2A10 \| solute carrier family 2 member 10 [Source:HGNC Symbol;Acc:HGNC:13444] \| -1.02415 \| \| HACD3 \| 3-hydroxyacyl-CoA dehydratase 3 [Source:HGNC Symbol;Acc:HGNC:24175] \| -1.02317 \| \| SPON2 \| spondin 2 [Source:HGNC Symbol;Acc:HGNC:11253] \| -1.02275 \| \| DNMT3B \| DNA methyltransferase 3 beta [Source:HGNC Symbol;Acc:HGNC:2979] \| -1.02234 \| \| SLC26A9 \| solute carrier family 26 member 9 [Source:HGNC Symbol;Acc:HGNC:14469] \| -1.01749 \| \| H19 \| H19, imprinted maternally expressed transcript [Source:HGNC Symbol;Acc:HGNC:4713] \| -1.01746 \| \| ACER3 \| alkaline ceramidase 3 [Source:HGNC Symbol;Acc:HGNC:16066] \| -1.01533 \| \| TMC6 \| transmembrane channel like 6 [Source:HGNC Symbol;Acc:HGNC:18021] \| -1.01461 \| \| ZNF117 \| zinc finger protein 117 [Source:HGNC Symbol;Acc:HGNC:12897] \| -1.01342 \| \| SCOC \| short coiled-coil protein [Source:HGNC Symbol;Acc:HGNC:20335] \| -1.01342 \| \| AQP7 \| aquaporin 7 [Source:HGNC Symbol;Acc:HGNC:640] \| -1.01342 \| \| USP49 \| ubiquitin specific peptidase 49 [Source:HGNC Symbol;Acc:HGNC:20078] \| -1.01341 \| \| MTTP \| microsomal triglyceride transfer protein [Source:HGNC Symbol;Acc:HGNC:7467] \| -1.0096 \| \| SLC16A13 \| solute carrier family 16 member 13 [Source:HGNC Symbol;Acc:HGNC:31037] \| -1.00932 \| \| NFIA \| nuclear factor I A [Source:HGNC Symbol;Acc:HGNC:7784] \| -1.00837 \| \| UBALD2 \| UBA like domain containing 2 [Source:HGNC Symbol;Acc:HGNC:28438] \| -1.00799 \| \| SLC24A1 \| solute carrier family 24 member 1 [Source:HGNC Symbol;Acc:HGNC:10975] \| -1.00641 \| \| HOOK1 \| hook microtubule tethering protein 1 [Source:HGNC Symbol;Acc:HGNC:19884] \| -1.00573 \| \| GMDS \| GDP-mannose 4,6-dehydratase [Source:HGNC Symbol;Acc:HGNC:4369] \| -1.00547 \| \| SCAMP1 \| secretory carrier membrane protein 1 [Source:HGNC Symbol;Acc:HGNC:10563] \| -1.00451 \| \| GCHFR \| GTP cyclohydrolase I feedback regulator [Source:HGNC Symbol;Acc:HGNC:4194] \| -1.00104 \| \| PLAT \| plasminogen activator, tissue type [Source:HGNC Symbol;Acc:HGNC:9051] \| -1.00086 \| \| ALB \| albumin [Source:HGNC Symbol;Acc:HGNC:399] \| -1.00077 \| \| GPCPD1 \| glycerophosphocholine phosphodiesterase 1 [Source:HGNC Symbol;Acc:HGNC:26957] \| -1.00025 \| |
| --- | --- | --- | --- | --- | --- | --- | --- | --- | --- | --- | --- | --- | --- | --- | --- | --- | --- | --- | --- | --- | --- | --- | --- | --- | --- | --- | --- | --- | --- | --- | --- | --- | --- | --- | --- | --- | --- | --- | --- | --- | --- | --- | --- | --- | --- | --- | --- | --- | --- | --- | --- | --- | --- | --- | --- | --- | --- | --- | --- | --- | --- | --- | --- | --- | --- | --- | --- | --- | --- | --- | --- | --- | --- | --- | --- | --- | --- | --- | --- | --- | --- | --- | --- | --- | --- | --- | --- | --- | --- | --- | --- | --- | --- | --- | --- | --- | --- | --- | --- | --- | --- | --- | --- | --- | --- | --- | --- | --- | --- | --- | --- | --- | --- | --- | --- | --- | --- | --- | --- | --- | --- | --- | --- | --- | --- | --- | --- | --- | --- | --- | --- | --- | --- | --- | --- | --- | --- | --- | --- | --- | --- | --- | --- | --- | --- | --- | --- | --- | --- | --- | --- | --- | --- | --- | --- | --- | --- | --- | --- | --- | --- | --- | --- | --- | --- | --- | --- | --- | --- | --- | --- | --- | --- | --- | --- | --- | --- | --- | --- | --- | --- | --- | --- | --- | --- | --- | --- | --- | --- | --- | --- | --- | --- | --- | --- | --- | --- | --- | --- | --- | --- | --- | --- | --- | --- | --- | --- | --- | --- | --- | --- | --- | --- | --- | --- | --- | --- | --- | --- | --- | --- | --- | --- | --- | --- | --- | --- | --- | --- | --- | --- | --- | --- | --- | --- | --- | --- | --- | --- | --- | --- | --- | --- | --- | --- | --- | --- | --- | --- | --- | --- | --- | --- | --- | --- | --- | --- | --- | --- | --- | --- | --- | --- | --- | --- | --- | --- | --- | --- | --- | --- | --- | --- | --- | --- | --- | --- | --- | --- | --- | --- | --- | --- | --- | --- | --- | --- | --- | --- | --- | --- | --- | --- | --- | --- | --- | --- | --- | --- | --- | --- | --- | --- | --- | --- | --- | --- | --- | --- | --- | --- | --- | --- | --- | --- | --- | --- | --- | --- | --- | --- | --- | --- | --- | --- | --- | --- | --- | --- | --- | --- | --- | --- | --- | --- | --- | --- | --- | --- | --- | --- | --- | --- | --- | --- | --- | --- | --- | --- | --- | --- | --- | --- | --- | --- | --- | --- | --- | --- | --- | --- | --- | --- | --- | --- | --- | --- | --- | --- | --- | --- | --- | --- | --- | --- | --- | --- | --- | --- | --- | --- | --- | --- | --- | --- | --- | --- | --- | --- | --- | --- | --- | --- | --- | --- | --- | --- | --- | --- | --- | --- | --- | --- | --- | --- | --- | --- | --- | --- | --- | --- | --- | --- | --- | --- | --- | --- | --- | --- | --- | --- | --- | --- | --- | --- | --- | --- | --- | --- | --- | --- | --- | --- | --- | --- | --- | --- | --- | --- | --- | --- | --- | --- | --- | --- | --- | --- | --- | --- | --- | --- | --- | --- | --- | --- | --- | --- | --- | --- | --- | --- | --- | --- | --- | --- | --- | --- | --- | --- | --- | --- | --- | --- | --- | --- | --- | --- | --- | --- | --- | --- | --- | --- | --- | --- | --- | --- | --- | --- | --- | --- | --- | --- | --- | --- | --- | --- | --- | --- | --- | --- | --- | --- | --- | --- | --- | --- | --- | --- | --- | --- | --- | --- | --- | --- | --- | --- | --- | --- | --- | --- | --- | --- | --- | --- | --- | --- | --- | --- | --- | --- | --- | --- | --- | --- | --- | --- | --- | --- | --- | --- | --- | --- | --- | --- | --- | --- | --- | --- | --- | --- | --- | --- | --- | --- | --- | --- | --- | --- | --- | --- | --- | --- | --- | --- | --- | --- | --- | --- | --- | --- | --- | --- | --- | --- | --- | --- | --- | --- | --- | --- | --- | --- | --- | --- | --- | --- | --- | --- | --- | --- | --- | --- | --- | --- | --- | --- | --- | --- | --- | --- | --- | --- | --- | --- | --- | --- | --- | --- | --- | --- | --- | --- | --- | --- | --- | --- | --- | --- | --- | --- | --- | --- | --- | --- | --- | --- | --- | --- | --- | --- | --- | --- | --- | --- | --- | --- | --- | --- | --- | --- | --- | --- | --- | --- | --- | --- | --- | --- | --- | --- | --- | --- | --- | --- | --- | --- | --- | --- | --- | --- | --- | --- | --- | --- | --- | --- | --- | --- | --- | --- | --- | --- | --- | --- | --- | --- | --- | --- | --- | --- | --- | --- | --- | --- | --- | --- | --- | --- | --- | --- | --- | --- | --- | --- | --- | --- | --- | --- | --- | --- | --- | --- | --- | --- | --- | --- | --- | --- | --- | --- | --- | --- | --- | --- | --- | --- | --- | --- | --- | --- | --- | --- | --- | --- | --- | --- | --- | --- | --- | --- | --- | --- | --- | --- | --- | --- | --- | --- | --- | --- | --- | --- | --- | --- | --- | --- | --- | --- | --- | --- | --- | --- | --- | --- | --- | --- | --- | --- | --- | --- | --- | --- | --- | --- | --- | --- | --- | --- | --- | --- | --- | --- | --- | --- | --- | --- | --- | --- | --- | --- | --- | --- | --- | --- | --- | --- | --- | --- | --- | --- | --- | --- | --- | --- | --- | --- | --- | --- | --- | --- | --- | --- | --- | --- | --- | --- | --- | --- | --- | --- | --- | --- | --- | --- | --- | --- | --- | --- | --- | --- | --- | --- | --- | --- | --- | --- | --- | --- | --- | --- | --- | --- | --- | --- | --- | --- | --- | --- | --- | --- | --- | --- | --- | --- | --- | --- | --- | --- | --- | --- | --- | --- | --- | --- | --- | --- | --- | --- | --- | --- | --- | --- | --- | --- | --- | --- | --- | --- | --- | --- | --- | --- | --- | --- | --- | --- | --- | --- | --- | --- | --- | --- | --- | --- | --- | --- | --- | --- | --- | --- | --- | --- | --- | --- | --- | --- | --- | --- | --- | --- | --- | --- | --- | --- | --- | --- | --- | --- | --- | --- | --- | --- | --- | --- | --- | --- | --- | --- | --- | --- | --- | --- | --- | --- | --- | --- | --- | --- | --- | --- | --- | --- | --- | --- | --- | --- | --- | --- | --- | --- | --- | --- | --- | --- | --- | --- | --- | --- | --- | --- | --- | --- | --- | --- | --- | --- | --- | --- | --- | --- | --- | --- | --- | --- | --- | --- | --- | --- | --- | --- | --- | --- | --- | --- | --- | --- | --- | --- | --- | --- | --- | --- | --- | --- | --- | --- | --- | --- | --- | --- | --- | --- | --- | --- | --- | --- | --- | --- | --- | --- | --- | --- | --- | --- | --- | --- | --- | --- | --- | --- | --- | --- | --- | --- | --- | --- | --- | --- | --- | --- | --- | --- | --- | --- | --- | --- | --- | --- | --- | --- | --- | --- | --- | --- | --- | --- | --- | --- | --- | --- | --- | --- | --- | --- | --- | --- | --- | --- | --- | --- | --- | --- | --- | --- | --- | --- | --- | --- | --- | --- | --- | --- | --- | --- | --- | --- | --- | --- | --- | --- | --- | --- | --- | --- | --- | --- | --- | --- | --- | --- | --- | --- | --- | --- | --- | --- | --- | --- | --- | --- | --- | --- | --- | --- | --- | --- | --- | --- | --- | --- | --- | --- | --- | --- | --- | --- | --- | --- | --- | --- | --- | --- | --- | --- | --- | --- | --- | --- | --- | --- | --- | --- | --- | --- | --- | --- | --- | --- | --- | --- | --- | --- | --- | --- | --- | --- | --- | --- | --- | --- | --- | --- | --- | --- | --- | --- | --- | --- | --- | --- | --- | --- | --- | --- | --- | --- | --- | --- | --- | --- | --- | --- | --- | --- | --- | --- | --- | --- | --- | --- | --- | --- | --- | --- | --- | --- | --- | --- | --- | --- | --- | --- | --- | --- | --- | --- | --- | --- | --- | --- | --- | --- | --- | --- | --- | --- | --- | --- | --- | --- | --- | --- | --- | --- | --- | --- | --- | --- | --- | --- | --- | --- | --- | --- | --- | --- | --- | --- | --- | --- | --- | --- | --- | --- | --- | --- | --- | --- | --- | --- | --- | --- | --- | --- | --- | --- | --- | --- | --- | --- | --- | --- | --- | --- | --- | --- | --- | --- | --- | --- | --- | --- | --- | --- | --- | --- | --- | --- | --- | --- | --- | --- | --- | --- | --- | --- | --- | --- | --- | --- | --- | --- | --- | --- | --- | --- | --- | --- | --- | --- | --- | --- | --- | --- | --- | --- | --- | --- | --- | --- | --- | --- | --- | --- | --- | --- | --- | --- | --- | --- | --- | --- | --- | --- | --- | --- | --- | --- | --- | --- | --- | --- | --- | --- | --- | --- | --- | --- | --- | --- | --- | --- | --- | --- | --- | --- | --- | --- | --- | --- | --- | --- | --- | --- | --- | --- | --- | --- | --- | --- | --- | --- | --- | --- | --- | --- | --- | --- | --- | --- | --- | --- | --- | --- | --- | --- | --- | --- | --- | --- | --- | --- | --- | --- | --- | --- | --- | --- | --- | --- | --- | --- | --- | --- | --- | --- | --- | --- | --- | --- | --- | --- |

**Supplementary Table S2 List of genes upregulated in HCCLM3-shZNF703 versus HCCLM3-shcontrol cells**

| Gene description log2FoldChange   \| FTH1P10 \| ferritin heavy chain 1 pseudogene 10 [Source:HGNC Symbol;Acc:HGNC:3980] \| 7.159072 \| \| --- \| --- \| --- \| \| RPS10-NUDT3 \| RPS10-NUDT3 readthrough [Source:HGNC Symbol;Acc:HGNC:49181] \| 6.352907 \| \| BMS1P2 \| BMS1, ribosome biogenesis factor pseudogene 2 [Source:HGNC Symbol;Acc:HGNC:23650] \| 5.629951 \| \| AL645608.7 \| novel transcript \| 5.308512 \| \| AC010323.1 \| novel transcript \| 5.115366 \| \| AC011448.1 \| readthrough between NDUFA13 and YJEFN3 \| 5.001532 \| \| AC093668.1 \| novel protein \| 4.940301 \| \| PCDHGC4 \| protocadherin gamma subfamily C, 4 [Source:HGNC Symbol;Acc:HGNC:8717] \| 4.308052 \| \| KBTBD11-OT1 \| KBTBD11 overlapping transcript 1 [Source:NCBI gene;Acc:104266957] \| 4.108213 \| \| PCDHGA9 \| protocadherin gamma subfamily A, 9 [Source:HGNC Symbol;Acc:HGNC:8707] \| 4.073603 \| \| ADAM1A \| ADAM metallopeptidase domain 1A (pseudogene) [Source:HGNC Symbol;Acc:HGNC:187] \| 3.794039 \| \| TMEM189-UBE2V1 \| TMEM189-UBE2V1 readthrough [Source:HGNC Symbol;Acc:HGNC:33521] \| 3.689206 \| \| AC025283.2 \| - \| 3.686541 \| \| GAPDHP72 \| glyceraldehyde-3-phosphate dehydrogenase pseudogene 72 [Source:HGNC Symbol;Acc:HGNC:22955] \| 3.686541 \| \| AC004080.3 \| HOXA10-HOXA9 readthrough \| 3.686541 \| \| AP000347.2 \| novel transcript \| 3.501094 \| \| FTCDNL1 \| formiminotransferase cyclodeaminase N-terminal like [Source:HGNC Symbol;Acc:HGNC:48661] \| 3.308778 \| \| AL359313.1 \| uncharacterized LOC153910 [Source:NCBI gene;Acc:153910] \| 3.308488 \| \| 4-Mar \| membrane associated ring-CH-type finger 4 [Source:HGNC Symbol;Acc:HGNC:29269] \| 3.292391 \| \| KCNK4-TEX40 \| KCNK4-TEX40 readthrough [Source:NCBI gene;Acc:106780802] \| 3.259597 \| \| C6orf222 \| chromosome 6 open reading frame 222 [Source:HGNC Symbol;Acc:HGNC:33769] \| 3.07433 \| \| AP000350.4 \| novel protein \| 3.074027 \| \| TICAM2 \| toll like receptor adaptor molecule 2 [Source:HGNC Symbol;Acc:HGNC:21354] \| 3.045473 \| \| LEXM \| lymphocyte expansion molecule [Source:HGNC Symbol;Acc:HGNC:26854] \| 2.986814 \| \| NEDD8-MDP1 \| NEDD8-MDP1 readthrough [Source:HGNC Symbol;Acc:HGNC:39551] \| 2.986814 \| \| AP000695.1 \| novel transcript \| 2.986814 \| \| AC006254.1 \| novel MUSTN1-ITIH4 readthrough \| 2.986814 \| \| AC005324.3 \| novel tripartite motif-containing 16 (TRIM16) and CMT1A duplicated region transcript 1 (CDRT1) protein \| 2.904488 \| \| C10orf126 \| - \| 2.893467 \| \| AC036214.3 \| novel protein, TPD52-MRPS28 readthrough \| 2.89333 \| \| ZNF672 \| zinc finger protein 672 [Source:HGNC Symbol;Acc:HGNC:26179] \| 2.856949 \| \| AC004678.1 \| novel pseudogene \| 2.793936 \| \| SENP3-EIF4A1 \| SENP3-EIF4A1 readthrough (NMD candidate) [Source:HGNC Symbol;Acc:HGNC:49182] \| 2.713201 \| \| ADM5 \| adrenomedullin 5 (putative) [Source:HGNC Symbol;Acc:HGNC:27293] \| 2.686905 \| \| AC087289.3 \| novel transcript \| 2.651252 \| \| FAM47E-STBD1 \| FAM47E-STBD1 readthrough [Source:HGNC Symbol;Acc:HGNC:44667] \| 2.614592 \| \| AL365205.1 \| novel transcript \| 2.577389 \| \| LINC02015 \| long intergenic non-protein coding RNA 2015 [Source:HGNC Symbol;Acc:HGNC:52850] \| 2.571555 \| \| LAMC2 \| laminin subunit gamma 2 [Source:HGNC Symbol;Acc:HGNC:6493] \| 2.551016 \| \| AC007240.1 \| novel transcript \| 2.530888 \| \| BCL2A1 \| BCL2 related protein A1 [Source:HGNC Symbol;Acc:HGNC:991] \| 2.510036 \| \| PDXP \| pyridoxal phosphatase [Source:HGNC Symbol;Acc:HGNC:30259] \| 2.485544 \| \| AL596223.1 \| uncharacterized LOC101928994 [Source:NCBI gene;Acc:101928994] \| 2.43585 \| \| AC091390.4 \| uncharacterized LOC100289561 [Source:NCBI gene;Acc:100289561] \| 2.427189 \| \| SH3PXD2A \| SH3 and PX domains 2A [Source:HGNC Symbol;Acc:HGNC:23664] \| 2.413698 \| \| PRKAR2A-AS1 \| PRKAR2A antisense RNA 1 [Source:HGNC Symbol;Acc:HGNC:40471] \| 2.412893 \| \| IL2RG \| interleukin 2 receptor subunit gamma [Source:HGNC Symbol;Acc:HGNC:6010] \| 2.408087 \| \| PRDM2 \| PR/SET domain 2 [Source:HGNC Symbol;Acc:HGNC:9347] \| 2.370821 \| \| DLK2 \| delta like non-canonical Notch ligand 2 [Source:HGNC Symbol;Acc:HGNC:21113] \| 2.365194 \| \| AMH \| anti-Mullerian hormone [Source:HGNC Symbol;Acc:HGNC:464] \| 2.333046 \| \| PMP22 \| peripheral myelin protein 22 [Source:HGNC Symbol;Acc:HGNC:9118] \| 2.310756 \| \| AC135050.2 \| novel protein, VKORC1 and PRSS53 readthrough \| 2.308633 \| \| MED26 \| mediator complex subunit 26 [Source:HGNC Symbol;Acc:HGNC:2376] \| 2.292391 \| \| YTHDF2 \| YTH N6-methyladenosine RNA binding protein 2 [Source:HGNC Symbol;Acc:HGNC:31675] \| 2.291546 \| \| THAP9 \| THAP domain containing 9 [Source:HGNC Symbol;Acc:HGNC:23192] \| 2.261567 \| \| AC010343.3 \| novel transcript \| 2.208973 \| \| HAUS2 \| HAUS augmin like complex subunit 2 [Source:HGNC Symbol;Acc:HGNC:25530] \| 2.187939 \| \| Z82186.1 \| uncharacterized LOC339685 [Source:NCBI gene;Acc:339685] \| 2.156491 \| \| AC018523.2 \| novel transcript \| 2.156491 \| \| MED29 \| mediator complex subunit 29 [Source:HGNC Symbol;Acc:HGNC:23074] \| 2.15099 \| \| GRIA3 \| glutamate ionotropic receptor AMPA type subunit 3 [Source:HGNC Symbol;Acc:HGNC:4573] \| 2.124044 \| \| MRFAP1 \| Morf4 family associated protein 1 [Source:HGNC Symbol;Acc:HGNC:24549] \| 2.099294 \| \| CS \| citrate synthase [Source:HGNC Symbol;Acc:HGNC:2422] \| 2.076697 \| \| GPRASP1 \| G protein-coupled receptor associated sorting protein 1 [Source:HGNC Symbol;Acc:HGNC:24834] \| 2.045502 \| \| IL1A \| interleukin 1 alpha [Source:HGNC Symbol;Acc:HGNC:5991] \| 2.041045 \| \| LINC00656 \| long intergenic non-protein coding RNA 656 [Source:HGNC Symbol;Acc:HGNC:27304] \| 2.037135 \| \| NT5C3AP1 \| 5'-nucleotidase, cytosolic IIIA pseudogene 1 [Source:HGNC Symbol;Acc:HGNC:18530] \| 2.016338 \| \| MROH6 \| maestro heat like repeat family member 6 [Source:HGNC Symbol;Acc:HGNC:27814] \| 2.007164 \| \| KCNAB3 \| potassium voltage-gated channel subfamily A regulatory beta subunit 3 [Source:HGNC Symbol;Acc:HGNC:6230] \| 1.986633 \| \| AC078850.1 \| novel transcript \| 1.986529 \| \| DEPDC1-AS1 \| DEPDC1 antisense RNA 1 [Source:HGNC Symbol;Acc:HGNC:50592] \| 1.986529 \| \| MDM4 \| MDM4, p53 regulator [Source:HGNC Symbol;Acc:HGNC:6974] \| 1.984014 \| \| PDPK1 \| 3-phosphoinositide dependent protein kinase 1 [Source:HGNC Symbol;Acc:HGNC:8816] \| 1.968131 \| \| BRINP2 \| BMP/retinoic acid inducible neural specific 2 [Source:HGNC Symbol;Acc:HGNC:13746] \| 1.958545 \| \| AL355987.3 \| novel transcript \| 1.948077 \| \| MHENCR \| melanoma highly expressed competing endogenous lncRNA for miR-425 and miR-489 [Source:HGNC Symbol;Acc:HGNC:53110] \| 1.927674 \| \| PTTG1IP \| PTTG1 interacting protein [Source:HGNC Symbol;Acc:HGNC:13524] \| 1.925434 \| \| AC068580.4 \| novel protein \| 1.922226 \| \| DGKH \| diacylglycerol kinase eta [Source:HGNC Symbol;Acc:HGNC:2854] \| 1.915195 \| \| GAGE1 \| G antigen 1 [Source:HGNC Symbol;Acc:HGNC:4098] \| 1.912605 \| \| GLDC \| glycine decarboxylase [Source:HGNC Symbol;Acc:HGNC:4313] \| 1.904121 \| \| AC145098.2 \| TEC \| 1.900823 \| \| LRRC37A3 \| leucine rich repeat containing 37 member A3 [Source:HGNC Symbol;Acc:HGNC:32427] \| 1.896064 \| \| ADIRF-AS1 \| ADIRF antisense RNA 1 [Source:HGNC Symbol;Acc:HGNC:45127] \| 1.890347 \| \| CCNT2 \| cyclin T2 [Source:HGNC Symbol;Acc:HGNC:1600] \| 1.889901 \| \| ZNF253 \| zinc finger protein 253 [Source:HGNC Symbol;Acc:HGNC:13497] \| 1.879671 \| \| DLG2 \| discs large MAGUK scaffold protein 2 [Source:HGNC Symbol;Acc:HGNC:2901] \| 1.861058 \| \| CHRM4 \| cholinergic receptor muscarinic 4 [Source:HGNC Symbol;Acc:HGNC:1953] \| 1.847146 \| \| AC006159.1 \| novel transcript \| 1.844529 \| \| AL137782.1 \| novel transcript \| 1.834613 \| \| FOSL2 \| FOS like 2, AP-1 transcription factor subunit [Source:HGNC Symbol;Acc:HGNC:3798] \| 1.833837 \| \| YJEFN3 \| YjeF N-terminal domain containing 3 [Source:HGNC Symbol;Acc:HGNC:24785] \| 1.829878 \| \| CYP1A1 \| cytochrome P450 family 1 subfamily A member 1 [Source:HGNC Symbol;Acc:HGNC:2595] \| 1.822281 \| \| CREB3L2 \| cAMP responsive element binding protein 3 like 2 [Source:HGNC Symbol;Acc:HGNC:23720] \| 1.815152 \| \| TXNIP \| thioredoxin interacting protein [Source:HGNC Symbol;Acc:HGNC:16952] \| 1.804395 \| \| CARMIL2 \| capping protein regulator and myosin 1 linker 2 [Source:HGNC Symbol;Acc:HGNC:27089] \| 1.794013 \| \| C3orf33 \| chromosome 3 open reading frame 33 [Source:HGNC Symbol;Acc:HGNC:26434] \| 1.793931 \| \| WDFY3-AS2 \| WDFY3 antisense RNA 2 [Source:HGNC Symbol;Acc:HGNC:21603] \| 1.777952 \| \| LINC02577 \| long intergenic non-protein coding RNA 2577 [Source:HGNC Symbol;Acc:HGNC:53749] \| 1.775447 \| \| AL031282.2 \| novel transcript, readthrough between SLC35E2 and CDK11A \| 1.752117 \| \| RGPD1 \| RANBP2-like and GRIP domain containing 1 [Source:HGNC Symbol;Acc:HGNC:32414] \| 1.745563 \| \| AL590004.3 \| novel transcript \| 1.739266 \| \| MYZAP \| myocardial zonula adherens protein [Source:HGNC Symbol;Acc:HGNC:43444] \| 1.7338 \| \| FRMD3 \| FERM domain containing 3 [Source:HGNC Symbol;Acc:HGNC:24125] \| 1.721264 \| \| ANKRD52 \| ankyrin repeat domain 52 [Source:HGNC Symbol;Acc:HGNC:26614] \| 1.719959 \| \| RGS22 \| regulator of G protein signaling 22 [Source:HGNC Symbol;Acc:HGNC:24499] \| 1.7124 \| \| CSRNP1 \| cysteine and serine rich nuclear protein 1 [Source:HGNC Symbol;Acc:HGNC:14300] \| 1.707603 \| \| LINC01006 \| long intergenic non-protein coding RNA 1006 [Source:HGNC Symbol;Acc:HGNC:48971] \| 1.69441 \| \| MUC1 \| mucin 1, cell surface associated [Source:HGNC Symbol;Acc:HGNC:7508] \| 1.684309 \| \| ZNF34 \| zinc finger protein 34 [Source:HGNC Symbol;Acc:HGNC:13098] \| 1.674634 \| \| TCP1P1 \| t-complex 1 pseudogene 1 [Source:HGNC Symbol;Acc:HGNC:11659] \| 1.664644 \| \| ZC3H11B \| zinc finger CCCH-type containing 11B [Source:HGNC Symbol;Acc:HGNC:25659] \| 1.622882 \| \| KLF16 \| Kruppel like factor 16 [Source:HGNC Symbol;Acc:HGNC:16857] \| 1.610611 \| \| ZNF559 \| zinc finger protein 559 [Source:HGNC Symbol;Acc:HGNC:28197] \| 1.606739 \| \| SLC6A6 \| solute carrier family 6 member 6 [Source:HGNC Symbol;Acc:HGNC:11052] \| 1.60531 \| \| DHRS9 \| dehydrogenase/reductase 9 [Source:HGNC Symbol;Acc:HGNC:16888] \| 1.594366 \| \| PRR22 \| proline rich 22 [Source:HGNC Symbol;Acc:HGNC:28354] \| 1.571571 \| \| RWDD2B \| RWD domain containing 2B [Source:HGNC Symbol;Acc:HGNC:1302] \| 1.571546 \| \| CCDC51 \| coiled-coil domain containing 51 [Source:HGNC Symbol;Acc:HGNC:25714] \| 1.565697 \| \| STX6 \| syntaxin 6 [Source:HGNC Symbol;Acc:HGNC:11441] \| 1.547246 \| \| RAB4B-EGLN2 \| RAB4B-EGLN2 readthrough (NMD candidate) [Source:HGNC Symbol;Acc:HGNC:44465] \| 1.544192 \| \| PPM1F \| protein phosphatase, Mg2+/Mn2+ dependent 1F [Source:HGNC Symbol;Acc:HGNC:19388] \| 1.540924 \| \| AR \| androgen receptor [Source:HGNC Symbol;Acc:HGNC:644] \| 1.507351 \| \| AC015813.2 \| novel transcript \| 1.505942 \| \| FGFBP1 \| fibroblast growth factor binding protein 1 [Source:HGNC Symbol;Acc:HGNC:19695] \| 1.502418 \| \| RHOQ \| ras homolog family member Q [Source:HGNC Symbol;Acc:HGNC:17736] \| 1.495896 \| \| PALLD \| palladin, cytoskeletal associated protein [Source:HGNC Symbol;Acc:HGNC:17068] \| 1.488563 \| \| ITGB1-DT \| ITGB1 divergent transcript [Source:HGNC Symbol;Acc:HGNC:53718] \| 1.484072 \| \| DPH1 \| diphthamide biosynthesis 1 [Source:HGNC Symbol;Acc:HGNC:3003] \| 1.478432 \| \| KLF6 \| Kruppel like factor 6 [Source:HGNC Symbol;Acc:HGNC:2235] \| 1.476432 \| \| KCNN3 \| potassium calcium-activated channel subfamily N member 3 [Source:HGNC Symbol;Acc:HGNC:6292] \| 1.476232 \| \| AC005394.2 \| SHC binding and spindle associated 1 pseudogene [Source:NCBI gene;Acc:100420587] \| 1.466543 \| \| ARID5A \| AT-rich interaction domain 5A [Source:HGNC Symbol;Acc:HGNC:17361] \| 1.45975 \| \| NOL4L \| nucleolar protein 4 like [Source:HGNC Symbol;Acc:HGNC:16106] \| 1.451006 \| \| IL10RB \| interleukin 10 receptor subunit beta [Source:HGNC Symbol;Acc:HGNC:5965] \| 1.448797 \| \| EP400P1 \| EP400 pseudogene 1 [Source:HGNC Symbol;Acc:HGNC:26602] \| 1.417913 \| \| TMEM35B \| transmembrane protein 35B [Source:HGNC Symbol;Acc:HGNC:40021] \| 1.41374 \| \| CTBP1-DT \| CTBP1 divergent transcript [Source:HGNC Symbol;Acc:HGNC:28307] \| 1.412849 \| \| ART4 \| ADP-ribosyltransferase 4 (Dombrock blood group) [Source:HGNC Symbol;Acc:HGNC:726] \| 1.411069 \| \| TSEN15 \| tRNA splicing endonuclease subunit 15 [Source:HGNC Symbol;Acc:HGNC:16791] \| 1.410445 \| \| FSIP2 \| fibrous sheath interacting protein 2 [Source:HGNC Symbol;Acc:HGNC:21675] \| 1.406226 \| \| CYP26B1 \| cytochrome P450 family 26 subfamily B member 1 [Source:HGNC Symbol;Acc:HGNC:20581] \| 1.399362 \| \| ZNF766 \| zinc finger protein 766 [Source:HGNC Symbol;Acc:HGNC:28063] \| 1.398998 \| \| AC011603.2 \| novel transcript, antisense to TUBA1B \| 1.395997 \| \| AC016026.1 \| novel transcript \| 1.387096 \| \| GFPT2 \| glutamine-fructose-6-phosphate transaminase 2 [Source:HGNC Symbol;Acc:HGNC:4242] \| 1.384735 \| \| AC002066.1 \| novel transcript \| 1.384615 \| \| RGPD2 \| RANBP2-like and GRIP domain containing 2 [Source:HGNC Symbol;Acc:HGNC:32415] \| 1.383469 \| \| LINC00973 \| long intergenic non-protein coding RNA 973 [Source:HGNC Symbol;Acc:HGNC:48868] \| 1.381439 \| \| ZNF24 \| zinc finger protein 24 [Source:HGNC Symbol;Acc:HGNC:13032] \| 1.379324 \| \| GGT8P \| gamma-glutamyltransferase 8 pseudogene [Source:HGNC Symbol;Acc:HGNC:33438] \| 1.373624 \| \| MAFF \| MAF bZIP transcription factor F [Source:HGNC Symbol;Acc:HGNC:6780] \| 1.368008 \| \| TXN \| thioredoxin [Source:HGNC Symbol;Acc:HGNC:12435] \| 1.354392 \| \| MOB4 \| MOB family member 4, phocein [Source:HGNC Symbol;Acc:HGNC:17261] \| 1.354033 \| \| IL32 \| interleukin 32 [Source:HGNC Symbol;Acc:HGNC:16830] \| 1.353695 \| \| ZNF17 \| zinc finger protein 17 [Source:HGNC Symbol;Acc:HGNC:12958] \| 1.350996 \| \| NUP50 \| nucleoporin 50 [Source:HGNC Symbol;Acc:HGNC:8065] \| 1.350429 \| \| SLC16A1-AS1 \| SLC16A1 antisense RNA 1 [Source:HGNC Symbol;Acc:HGNC:49445] \| 1.347305 \| \| FAM102A \| family with sequence similarity 102 member A [Source:HGNC Symbol;Acc:HGNC:31419] \| 1.346151 \| \| ARMT1 \| acidic residue methyltransferase 1 [Source:HGNC Symbol;Acc:HGNC:17872] \| 1.343811 \| \| TMEM33 \| transmembrane protein 33 [Source:HGNC Symbol;Acc:HGNC:25541] \| 1.336392 \| \| IL18 \| interleukin 18 [Source:HGNC Symbol;Acc:HGNC:5986] \| 1.331962 \| \| ZNF343 \| zinc finger protein 343 [Source:HGNC Symbol;Acc:HGNC:16017] \| 1.330872 \| \| SNHG10 \| small nucleolar RNA host gene 10 [Source:HGNC Symbol;Acc:HGNC:27510] \| 1.330551 \| \| TYW3 \| tRNA-yW synthesizing protein 3 homolog [Source:HGNC Symbol;Acc:HGNC:24757] \| 1.328894 \| \| HYPK \| huntingtin interacting protein K [Source:HGNC Symbol;Acc:HGNC:18418] \| 1.324792 \| \| CCDC93 \| coiled-coil domain containing 93 [Source:HGNC Symbol;Acc:HGNC:25611] \| 1.321022 \| \| ADGRF4 \| adhesion G protein-coupled receptor F4 [Source:HGNC Symbol;Acc:HGNC:19011] \| 1.320987 \| \| IPO8P1 \| importin 8 pseudogene 1 [Source:HGNC Symbol;Acc:HGNC:41955] \| 1.319158 \| \| AC116366.3 \| novel protein \| 1.316282 \| \| SELENON \| selenoprotein N [Source:HGNC Symbol;Acc:HGNC:15999] \| 1.31489 \| \| ERCC4 \| ERCC excision repair 4, endonuclease catalytic subunit [Source:HGNC Symbol;Acc:HGNC:3436] \| 1.314633 \| \| AC114490.2 \| novel transcript, ZMYM6-ZMYM6NB readthrough \| 1.308505 \| \| IL4R \| interleukin 4 receptor [Source:HGNC Symbol;Acc:HGNC:6015] \| 1.304192 \| \| MBP \| myelin basic protein [Source:HGNC Symbol;Acc:HGNC:6925] \| 1.29879 \| \| AL162582.1 \| uncharacterized LOC105378088 [Source:NCBI gene;Acc:105378088] \| 1.297579 \| \| NPY6R \| neuropeptide Y receptor Y6 (pseudogene) [Source:HGNC Symbol;Acc:HGNC:7959] \| 1.288468 \| \| LAMB3 \| laminin subunit beta 3 [Source:HGNC Symbol;Acc:HGNC:6490] \| 1.285283 \| \| DAZAP2 \| DAZ associated protein 2 [Source:HGNC Symbol;Acc:HGNC:2684] \| 1.284186 \| \| AC134772.2 \| - \| 1.277464 \| \| ZNF181 \| zinc finger protein 181 [Source:HGNC Symbol;Acc:HGNC:12971] \| 1.273458 \| \| AP002884.1 \| uncharacterized LOC283140 [Source:NCBI gene;Acc:283140] \| 1.268963 \| \| ELFN2 \| extracellular leucine rich repeat and fibronectin type III domain containing 2 [Source:HGNC Symbol;Acc:HGNC:29396] \| 1.267423 \| \| NFE2L1 \| nuclear factor, erythroid 2 like 1 [Source:HGNC Symbol;Acc:HGNC:7781] \| 1.247436 \| \| EEF1A2 \| eukaryotic translation elongation factor 1 alpha 2 [Source:HGNC Symbol;Acc:HGNC:3192] \| 1.245647 \| \| RN7SK \| RNA, 7SK small nuclear [Source:HGNC Symbol;Acc:HGNC:10037] \| 1.23933 \| \| KDR \| kinase insert domain receptor [Source:HGNC Symbol;Acc:HGNC:6307] \| 1.236216 \| \| CCDC180 \| coiled-coil domain containing 180 [Source:HGNC Symbol;Acc:HGNC:29303] \| 1.234515 \| \| FLNC \| filamin C [Source:HGNC Symbol;Acc:HGNC:3756] \| 1.226875 \| \| BCL2L2-PABPN1 \| BCL2L2-PABPN1 readthrough [Source:HGNC Symbol;Acc:HGNC:42959] \| 1.226784 \| \| SLC35A2 \| solute carrier family 35 member A2 [Source:HGNC Symbol;Acc:HGNC:11022] \| 1.225312 \| \| FOXM1 \| forkhead box M1 [Source:HGNC Symbol;Acc:HGNC:3818] \| 1.221309 \| \| CASP10 \| caspase 10 [Source:HGNC Symbol;Acc:HGNC:1500] \| 1.212633 \| \| WIPF2 \| WAS/WASL interacting protein family member 2 [Source:HGNC Symbol;Acc:HGNC:30923] \| 1.210312 \| \| COMMD3-BMI1 \| COMMD3-BMI1 readthrough [Source:HGNC Symbol;Acc:HGNC:48326] \| 1.208978 \| \| AC091173.1 \| novel transcript \| 1.208977 \| \| KRT80 \| keratin 80 [Source:HGNC Symbol;Acc:HGNC:27056] \| 1.208973 \| \| UNC13D \| unc-13 homolog D [Source:HGNC Symbol;Acc:HGNC:23147] \| 1.208966 \| \| F11R \| F11 receptor [Source:HGNC Symbol;Acc:HGNC:14685] \| 1.204623 \| \| LINC02535 \| long intergenic non-protein coding RNA 2535 [Source:HGNC Symbol;Acc:HGNC:53569] \| 1.200684 \| \| ZMYM5 \| zinc finger MYM-type containing 5 [Source:HGNC Symbol;Acc:HGNC:13029] \| 1.193015 \| \| GBP1 \| guanylate binding protein 1 [Source:HGNC Symbol;Acc:HGNC:4182] \| 1.192743 \| \| SC5D \| sterol-C5-desaturase [Source:HGNC Symbol;Acc:HGNC:10547] \| 1.190871 \| \| ZNFX1 \| zinc finger NFX1-type containing 1 [Source:HGNC Symbol;Acc:HGNC:29271] \| 1.189372 \| \| BHLHE41 \| basic helix-loop-helix family member e41 [Source:HGNC Symbol;Acc:HGNC:16617] \| 1.188198 \| \| FAM53C \| family with sequence similarity 53 member C [Source:HGNC Symbol;Acc:HGNC:1336] \| 1.187164 \| \| TLDC2 \| TBC/LysM-associated domain containing 2 [Source:HGNC Symbol;Acc:HGNC:16112] \| 1.184847 \| \| FGF2 \| fibroblast growth factor 2 [Source:HGNC Symbol;Acc:HGNC:3676] \| 1.184304 \| \| GLIS2 \| GLIS family zinc finger 2 [Source:HGNC Symbol;Acc:HGNC:29450] \| 1.184195 \| \| VDR \| vitamin D receptor [Source:HGNC Symbol;Acc:HGNC:12679] \| 1.178825 \| \| PPAN-P2RY11 \| PPAN-P2RY11 readthrough [Source:HGNC Symbol;Acc:HGNC:33526] \| 1.169739 \| \| BCL2L15 \| BCL2 like 15 [Source:HGNC Symbol;Acc:HGNC:33624] \| 1.169315 \| \| NRM \| nurim [Source:HGNC Symbol;Acc:HGNC:8003] \| 1.166396 \| \| STIMATE \| STIM activating enhancer [Source:HGNC Symbol;Acc:HGNC:30526] \| 1.161454 \| \| PCDHGB5 \| protocadherin gamma subfamily B, 5 [Source:HGNC Symbol;Acc:HGNC:8712] \| 1.160408 \| \| MIR100HG \| mir-100-let-7a-2-mir-125b-1 cluster host gene [Source:HGNC Symbol;Acc:HGNC:39522] \| 1.160408 \| \| S100A2 \| S100 calcium binding protein A2 [Source:HGNC Symbol;Acc:HGNC:10492] \| 1.153039 \| \| ZNF774 \| zinc finger protein 774 [Source:HGNC Symbol;Acc:HGNC:33108] \| 1.145789 \| \| LYRM4 \| LYR motif containing 4 [Source:HGNC Symbol;Acc:HGNC:21365] \| 1.144835 \| \| ZNF84 \| zinc finger protein 84 [Source:HGNC Symbol;Acc:HGNC:13159] \| 1.143784 \| \| TRIM56 \| tripartite motif containing 56 [Source:HGNC Symbol;Acc:HGNC:19028] \| 1.142749 \| \| LIPG \| lipase G, endothelial type [Source:HGNC Symbol;Acc:HGNC:6623] \| 1.142364 \| \| METTL16 \| methyltransferase like 16 [Source:HGNC Symbol;Acc:HGNC:28484] \| 1.142191 \| \| TCTA \| T cell leukemia translocation altered [Source:HGNC Symbol;Acc:HGNC:11692] \| 1.137134 \| \| EXPH5 \| exophilin 5 [Source:HGNC Symbol;Acc:HGNC:30578] \| 1.136334 \| \| NLGN3 \| neuroligin 3 [Source:HGNC Symbol;Acc:HGNC:14289] \| 1.134662 \| \| KLF4 \| Kruppel like factor 4 [Source:HGNC Symbol;Acc:HGNC:6348] \| 1.133837 \| \| AP000439.2 \| novel transcript \| 1.127057 \| \| AL161431.1 \| novel transcript \| 1.120435 \| \| DNER \| delta/notch like EGF repeat containing [Source:HGNC Symbol;Acc:HGNC:24456] \| 1.119834 \| \| JRK \| Jrk helix-turn-helix protein [Source:HGNC Symbol;Acc:HGNC:6199] \| 1.119479 \| \| MED31 \| mediator complex subunit 31 [Source:HGNC Symbol;Acc:HGNC:24260] \| 1.116344 \| \| ARRDC4 \| arrestin domain containing 4 [Source:HGNC Symbol;Acc:HGNC:28087] \| 1.115286 \| \| TDP2 \| tyrosyl-DNA phosphodiesterase 2 [Source:HGNC Symbol;Acc:HGNC:17768] \| 1.114285 \| \| DOCK10 \| dedicator of cytokinesis 10 [Source:HGNC Symbol;Acc:HGNC:23479] \| 1.108553 \| \| MIEN1 \| migration and invasion enhancer 1 [Source:HGNC Symbol;Acc:HGNC:28230] \| 1.107499 \| \| LCA5 \| LCA5, lebercilin [Source:HGNC Symbol;Acc:HGNC:31923] \| 1.103386 \| \| NEK10 \| NIMA related kinase 10 [Source:HGNC Symbol;Acc:HGNC:18592] \| 1.102063 \| \| SLC46A1 \| solute carrier family 46 member 1 [Source:HGNC Symbol;Acc:HGNC:30521] \| 1.10135 \| \| TSN \| translin [Source:HGNC Symbol;Acc:HGNC:12379] \| 1.100977 \| \| TMEM79 \| transmembrane protein 79 [Source:HGNC Symbol;Acc:HGNC:28196] \| 1.100794 \| \| KIAA1586 \| KIAA1586 [Source:HGNC Symbol;Acc:HGNC:21360] \| 1.100137 \| \| AC092718.3 \| novel protein \| 1.097613 \| \| PPFIBP2 \| PPFIA binding protein 2 [Source:HGNC Symbol;Acc:HGNC:9250] \| 1.094434 \| \| KBTBD4 \| kelch repeat and BTB domain containing 4 [Source:HGNC Symbol;Acc:HGNC:23761] \| 1.092566 \| \| LINC00460 \| long intergenic non-protein coding RNA 460 [Source:HGNC Symbol;Acc:HGNC:42809] \| 1.091372 \| \| TIAF1 \| TGFB1-induced anti-apoptotic factor 1 [Source:HGNC Symbol;Acc:HGNC:11803] \| 1.088932 \| \| CCDC68 \| coiled-coil domain containing 68 [Source:HGNC Symbol;Acc:HGNC:24350] \| 1.086106 \| \| AC006538.1 \| novel transcript \| 1.083866 \| \| SLC38A5 \| solute carrier family 38 member 5 [Source:HGNC Symbol;Acc:HGNC:18070] \| 1.083678 \| \| WSCD1 \| WSC domain containing 1 [Source:HGNC Symbol;Acc:HGNC:29060] \| 1.078745 \| \| AC125807.2 \| novel transcript \| 1.07848 \| \| AL133352.1 \| NADH dehydrogenase (ubiquinone) 1 beta subcomplex, 8, 19kDa (NDUFB8) and SEC31 homolog B (S. cerevisiae) (SEC31B) readthrough \| 1.073262 \| \| SLPI \| secretory leukocyte peptidase inhibitor [Source:HGNC Symbol;Acc:HGNC:11092] \| 1.068105 \| \| SYNPO \| synaptopodin [Source:HGNC Symbol;Acc:HGNC:30672] \| 1.065671 \| \| LINC01559 \| long intergenic non-protein coding RNA 1559 [Source:HGNC Symbol;Acc:HGNC:26598] \| 1.065428 \| \| CDCA7 \| cell division cycle associated 7 [Source:HGNC Symbol;Acc:HGNC:14628] \| 1.058726 \| \| RIMKLB \| ribosomal modification protein rimK like family member B [Source:HGNC Symbol;Acc:HGNC:29228] \| 1.050688 \| \| NTN4 \| netrin 4 [Source:HGNC Symbol;Acc:HGNC:13658] \| 1.044596 \| \| RNMT \| RNA guanine-7 methyltransferase [Source:HGNC Symbol;Acc:HGNC:10075] \| 1.031569 \| \| ZBTB49 \| zinc finger and BTB domain containing 49 [Source:HGNC Symbol;Acc:HGNC:19883] \| 1.030287 \| \| NIPAL1 \| NIPA like domain containing 1 [Source:HGNC Symbol;Acc:HGNC:27194] \| 1.026105 \| \| AC087632.1 \| novel transcript \| 1.023102 \| \| WDR20 \| WD repeat domain 20 [Source:HGNC Symbol;Acc:HGNC:19667] \| 1.021557 \| \| ABCB11 \| ATP binding cassette subfamily B member 11 [Source:HGNC Symbol;Acc:HGNC:42] \| 1.019744 \| \| EID3 \| EP300 interacting inhibitor of differentiation 3 [Source:HGNC Symbol;Acc:HGNC:32961] \| 1.01592 \| \| LINC00857 \| long intergenic non-protein coding RNA 857 [Source:HGNC Symbol;Acc:HGNC:45114] \| 1.01273 \| \| MED18 \| mediator complex subunit 18 [Source:HGNC Symbol;Acc:HGNC:25944] \| 1.012571 \| \| MB21D2 \| Mab-21 domain containing 2 [Source:HGNC Symbol;Acc:HGNC:30438] \| 1.010815 \| \| ZCWPW1 \| zinc finger CW-type and PWWP domain containing 1 [Source:HGNC Symbol;Acc:HGNC:23486] \| 1.008436 \| \| ZNF579 \| zinc finger protein 579 [Source:HGNC Symbol;Acc:HGNC:26646] \| 1.005194 \| \| SPRTN \| SprT-like N-terminal domain [Source:HGNC Symbol;Acc:HGNC:25356] \| 1.004138 \| \| AC009093.9 \| RRN3 RNA polymerase I transcription factor homolog (S. cerevisiae) (RRN3) pseudogene \| 1.003847 \| |
| --- | --- | --- | --- | --- | --- | --- | --- | --- | --- | --- | --- | --- | --- | --- | --- | --- | --- | --- | --- | --- | --- | --- | --- | --- | --- | --- | --- | --- | --- | --- | --- | --- | --- | --- | --- | --- | --- | --- | --- | --- | --- | --- | --- | --- | --- | --- | --- | --- | --- | --- | --- | --- | --- | --- | --- | --- | --- | --- | --- | --- | --- | --- | --- | --- | --- | --- | --- | --- | --- | --- | --- | --- | --- | --- | --- | --- | --- | --- | --- | --- | --- | --- | --- | --- | --- | --- | --- | --- | --- | --- | --- | --- | --- | --- | --- | --- | --- | --- | --- | --- | --- | --- | --- | --- | --- | --- | --- | --- | --- | --- | --- | --- | --- | --- | --- | --- | --- | --- | --- | --- | --- | --- | --- | --- | --- | --- | --- | --- | --- | --- | --- | --- | --- | --- | --- | --- | --- | --- | --- | --- | --- | --- | --- | --- | --- | --- | --- | --- | --- | --- | --- | --- | --- | --- | --- | --- | --- | --- | --- | --- | --- | --- | --- | --- | --- | --- | --- | --- | --- | --- | --- | --- | --- | --- | --- | --- | --- | --- | --- | --- | --- | --- | --- | --- | --- | --- | --- | --- | --- | --- | --- | --- | --- | --- | --- | --- | --- | --- | --- | --- | --- | --- | --- | --- | --- | --- | --- | --- | --- | --- | --- | --- | --- | --- | --- | --- | --- | --- | --- | --- | --- | --- | --- | --- | --- | --- | --- | --- | --- | --- | --- | --- | --- | --- | --- | --- | --- | --- | --- | --- | --- | --- | --- | --- | --- | --- | --- | --- | --- | --- | --- | --- | --- | --- | --- | --- | --- | --- | --- | --- | --- | --- | --- | --- | --- | --- | --- | --- | --- | --- | --- | --- | --- | --- | --- | --- | --- | --- | --- | --- | --- | --- | --- | --- | --- | --- | --- | --- | --- | --- | --- | --- | --- | --- | --- | --- | --- | --- | --- | --- | --- | --- | --- | --- | --- | --- | --- | --- | --- | --- | --- | --- | --- | --- | --- | --- | --- | --- | --- | --- | --- | --- | --- | --- | --- | --- | --- | --- | --- | --- | --- | --- | --- | --- | --- | --- | --- | --- | --- | --- | --- | --- | --- | --- | --- | --- | --- | --- | --- | --- | --- | --- | --- | --- | --- | --- | --- | --- | --- | --- | --- | --- | --- | --- | --- | --- | --- | --- | --- | --- | --- | --- | --- | --- | --- | --- | --- | --- | --- | --- | --- | --- | --- | --- | --- | --- | --- | --- | --- | --- | --- | --- | --- | --- | --- | --- | --- | --- | --- | --- | --- | --- | --- | --- | --- | --- | --- | --- | --- | --- | --- | --- | --- | --- | --- | --- | --- | --- | --- | --- | --- | --- | --- | --- | --- | --- | --- | --- | --- | --- | --- | --- | --- | --- | --- | --- | --- | --- | --- | --- | --- | --- | --- | --- | --- | --- | --- | --- | --- | --- | --- | --- | --- | --- | --- | --- | --- | --- | --- | --- | --- | --- | --- | --- | --- | --- | --- | --- | --- | --- | --- | --- | --- | --- | --- | --- | --- | --- | --- | --- | --- | --- | --- | --- | --- | --- | --- | --- | --- | --- | --- | --- | --- | --- | --- | --- | --- | --- | --- | --- | --- | --- | --- | --- | --- | --- | --- | --- | --- | --- | --- | --- | --- | --- | --- | --- | --- | --- | --- | --- | --- | --- | --- | --- | --- | --- | --- | --- | --- | --- | --- | --- | --- | --- | --- | --- | --- | --- | --- | --- | --- | --- | --- | --- | --- | --- | --- | --- | --- | --- | --- | --- | --- | --- | --- | --- | --- | --- | --- | --- | --- | --- | --- | --- | --- | --- | --- | --- | --- | --- | --- | --- | --- | --- | --- | --- | --- | --- | --- | --- | --- | --- | --- | --- | --- | --- | --- | --- | --- | --- | --- | --- | --- | --- | --- | --- | --- | --- | --- | --- | --- | --- | --- | --- | --- | --- | --- | --- | --- | --- | --- | --- | --- | --- | --- | --- | --- | --- | --- | --- | --- | --- | --- | --- | --- | --- | --- | --- | --- | --- | --- | --- | --- | --- | --- | --- | --- | --- | --- | --- | --- | --- | --- | --- | --- | --- | --- | --- | --- | --- | --- | --- | --- | --- | --- | --- | --- | --- | --- | --- | --- | --- | --- | --- | --- | --- | --- | --- | --- | --- | --- | --- | --- | --- | --- | --- | --- | --- | --- | --- | --- | --- | --- | --- | --- | --- | --- | --- | --- | --- | --- | --- | --- | --- | --- | --- | --- | --- | --- | --- | --- | --- | --- | --- | --- | --- | --- | --- | --- | --- | --- | --- | --- | --- | --- | --- | --- | --- | --- | --- | --- | --- | --- | --- | --- | --- | --- | --- | --- | --- | --- | --- | --- | --- | --- | --- | --- | --- | --- | --- | --- | --- | --- | --- | --- | --- | --- | --- | --- | --- | --- | --- | --- | --- | --- | --- | --- | --- | --- | --- | --- | --- | --- | --- | --- | --- | --- | --- | --- | --- | --- | --- | --- | --- | --- | --- | --- | --- | --- | --- | --- | --- | --- | --- | --- | --- | --- | --- | --- | --- | --- | --- | --- | --- | --- | --- | --- | --- | --- | --- | --- | --- | --- | --- | --- | --- | --- | --- | --- | --- | --- | --- | --- | --- | --- | --- | --- | --- | --- |
