# Supplementary material for: Zinc finger protein 703 induces EMT and sorafenib resistance in hepatocellular carcinoma by transactivating CLDN4 expression
Source: Cell Death Dis. 2020 Apr 8;11(4):225. doi: 10.1038/s41419-020-2422-3 (PMC7142083; doi:10.1038/s41419-020-2422-3)
Supplement: Supplementary file 3 — Supplementary Table S3 [file 41419_2020_2422_MOESM3_ESM.docx]

| **Supplementary Table S3 Univariate and multivariate analyses of ZNF703 expression and overall survival** | | | | | | | |
| --- | --- | --- | --- | --- | --- | --- | --- |
| Variables | Univariate analysis | | |  | Multivariate analysis | | |
|  | *P* | HR | 95%CI |  | *P* | HR | 95%CI |
| Age |  |  |  |  |  |  |  |
| ≤50 vs. >50 years | 0.692 | 0.954 | 0.757-1.203 |  |  |  |  |
| Gender |  |  |  |  |  |  |  |
| Male vs. female | 0.952 | 1.010 | 0.740-1.377 |  |  |  |  |
| HBsAg |  |  |  |  |  |  |  |
| positive vs.negative | 0.358 | 0.888 | 0.689-1.144 |  |  |  |  |
| Serum AFP |  |  |  |  |  |  |  |
| ≤20 vs.>20ng/ml | 0.228 | 0.875 | 0.705-1.087 |  |  |  |  |
| Cirrhosis |  |  |  |  |  |  |  |
| yes vs.no | 0.474 | 0.877 | 0.612-1.256 |  |  |  |  |
| Tumor differentiation | 0.020 | 1.276 | 0.902-1.805 |  | 0.443 | 1.225 | 0.877-1.712 |
| Tumor size |  |  |  |  |  |  |  |
| ≤5 vs.>5 cm | 0.003 | 1.329 | 1.000-1.766 |  | 0.239 | 1.116 | 0.846-1.473 |
| Tumor number |  |  |  |  |  |  |  |
| single vs.multiple | 0.012 | 1.036 | 0.648-1.656 |  | 0.451 | 0.906 | 0.727-1.130 |
| Tumor encapsulation |  |  |  |  |  |  |  |
| absent vs.present | 0.000 | 0.524 | 0.410-0.670 |  | 0.002 | 0.611 | 0.349-1.070 |
| Vascular invasion |  |  |  |  |  |  |  |
| yes vs.no | 0.000 | 2.020 | 1.130-3.612 |  | 0.197 | 1.113 | 0.882-1.406 |
| LNM |  |  |  |  |  |  |  |
| yes vs.no | 0.020 | 1.660 | 1.176-2.343 |  | 0.271 | 1.276 | 0.902-1.805 |
| TNM |  |  |  |  |  |  |  |
| I-II vs.III-IV | 0.000 | 2.093 | 1.720-2.546 |  | 0.087 | 1.225 | 0.877-1.712 |
| ZNF703 expression |  |  |  |  |  |  |  |
| low vs.high | 0.000 | 0.566 | 0.434-0.738 |  | 0.000 | 1.036 | 0.648-1.656 |

Abbreviation: AFP alpha-fetoprotein,LNM lymph node metastasis, HBsAg hepatitis B surface antigen, HR hazard ratio, CI confidence interval
